# Serum concentrations of legacy, alternative, and precursor per- and polyfluoroalkyl substances: a descriptive analysis of premenopausal participants in the Canadian CARTaGENE cohort

**DOI:** 10.1186/s12940-026-01326-3

**Published:** 2026-08-19

**Authors:** Michael M. Borghese, Rebecca Lelievre, Sara Packull-McCormick, Jillian Ashley-Martin, Maria P. Velez, Nolwenn Noisel, Mengting Xu, Jennifer E. Bruin, Tyler Pollock, Annie St-Amand

**Affiliations:** 1 Environmental Health Science and Research Bureau, Health Canada, Ottawa, Canada; 2 Department of Obstetrics and Gynecology, Research Institute McGill University Health Centre, McGill University, Montréal, Canada; 3 Département de Santé Environnementale et Santé au Travail, Université de Montréal, Montréal, Canada; 4 Centre de Recherche en Santé Publique (CReSP), Montréal, Canada; 5 CARTaGENE, Sainte-Justine University Hospital Research Center, Montréal, Canada; 6 Department of Biology, Institute of Biochemistry, Carleton University, Ottawa, Canada

**Keywords:** Biomonitoring, PFAS, Perfluorinated substances, Environmental chemicals, Perfluoroalkyl substances

## Abstract

**Background:**

Human biomonitoring studies of per- and polyfluoroalkyl substances (PFAS) have typically focused on legacy PFAS. Data for alternative and precursor PFAS are emerging, but are still scarce. These data are critical for assessing exposure and human health risk.

**Methods:**

We measured serum concentrations of 40 PFAS among 2,775 premenopausal participants from the CARTaGENE cohort (Phase A: 2009–2010; Phase B: 2013–2014). We examined differences in geometric mean serum concentrations of PFAS with > 60% detection, as well as the sum of 7 PFAS (Σ7PFAS), across categories of sociodemographic, lifestyle, and reproductive history characteristics.

**Results:**

Eight PFAS were detected in > 60% of participants. Geometric mean serum concentrations ranged from 0.03 µg/L (PFHpS and MeFOSAA) to 4.25 µg/L for PFOS and 8.00 µg/L for Σ7PFAS. The largest differences in serum PFAS concentrations were observed across reproductive history characteristics. Concentrations of PFOS, PFNA, PFOA, PFHxS, and the Σ7PFAS were monotonically lower with each additional child birthed, higher with increasing time since last pregnancy, and lower among those with a history of breastfeeding. The PFAS reducing effect of parity appeared to outweigh the bioaccumulative effect of age for most legacy PFAS. Concentrations of PFOS, PFNA, PFOA, PFHxS, PFDA, PFUnA and the Σ7PFAS were lower among participants with a reported household income below the Canadian low-income cut-off. Several alternative and precursor PFAS were detected in this population, including sulfonamidoacetic acids, sulfonamides, fluorotelomers, and fluoroethers, but serum concentrations were relatively low, with 95^th^ percentiles < 0.5 µg/L.

**Conclusions:**

This work contributes to expanding human biomonitoring data beyond legacy PFAS, and our findings suggest that exposure to low levels of some alternative and precursor PFAS may be widespread in Canada. These results also reinforce the role of pregnancy and breastfeeding as excretion pathways for legacy PFAS and support previous investigations demonstrating that exposure to some PFAS may be lower amongst financially disadvantaged populations.

**Supplementary Information:**

The online version contains supplementary material available at https://doi.org/10.1186/s12940-026-01326-3.

## Introduction

Per- and polyfluoroalkyl substances (PFAS) are a class of persistent chemicals commonly used in many household and consumer goods [1–4]. Exposure to these chemicals is ubiquitous in human populations around the world [5–7]; sources of exposure in the general population include food packaging, cosmetics, drinking water, and house dust [8], among others. The scientific evidence to date broadly supports deleterious associations between exposure to legacy PFAS – primarily PFOS (perfluorooctane sulfonate) and PFOA (perfluorooctanoic acid) – and health effects in humans [4, 9–12]. In Canada, risk management measures for PFOS, PFOA, and other long-chain perfluoroalkyl carboxylic acids have been in place since 2008/2010 [4], and their manufacture and use has been prohibited or restricted since 2016 [13]. In line with other human biomonitoring studies around the world [6, 14, 15], plasma concentrations of these legacy PFAS declined by 50–60% in Canada between 2007 and 2017 [16].

As a result of the risk management actions in Canada, the United States, and elsewhere around the world, these legacy PFAS have largely been phased out of production in favor of alternative or precursor PFAS [4, 17, 18]. There is limited information on the potential exposure and health effects of alternative and precursor PFAS, though some evidence is emerging [19–22]. Several of these PFAS have been detected with increasing concentration over time in Canadian freshwater [23] and wastewater [24] environments since 2009. A previous analysis conducted by our group using 2018–2021 data from non-pregnant adult females in the MIREC-ENDO study suggested that contemporary exposure to some alternative and precursor PFAS may be widespread at low levels [21]. Descriptive analyses of historical exposures are also needed to understand potential cumulative risks to human health and to evaluate the impacts of risk management actions over time. Such descriptive analyses allow for the characterization of exposure across a range of sociodemographic, lifestyle, and reproductive factors and can help to identify populations that may be disproportionately impacted by exposure to PFAS.

Serum concentrations of legacy PFAS have been shown to decline with increasing number of pregnancies (i.e., parity) [21, 25, 26] due at least in part to transplacental transfer [27, 28] as well as lactation [27–29]. The PFAS reducing effect of parity appears to contrast with the notion that legacy PFAS tend to bioaccumulate with increasing age because of their long half-lives [11] as well as age-related reductions in serum albumin concentrations and kidney function [30]. To our knowledge, the joint effect of parity and age on PFAS concentrations has not been directly investigated. Much of the evidence for parity as a determinant of PFAS serum concentrations is derived from pregnancy cohorts [21, 25], which by design exclude nulliparous women. Comparing the effect of age on serum PFAS concentrations among nulliparous and multiparous women could help disentangle these apparently opposing effects. Menstruation is another prominent pathway of PFAS excretion [31] and heterogeneity in menopausal status may obscure analyses of determinants of exposure, particularly for factors such as age and parity. Restricting analyses to premenopausal adults would provide a more physiologically homogeneous population to elucidate the determinants of PFAS body burden. In this paper, we present a descriptive analysis of serum concentrations of 40 legacy, alternative, and precursor PFAS among premenopausal adults from the Quebec-based CARTaGENE cohort. Our objective was to describe serum concentrations of these PFAS and to examine patterns of exposure according to sociodemographic, lifestyle, and reproductive history characteristics.

## Methods

### Study participants

CARTaGENE is a prospective population cohort study in Québec, Canada; detailed information on the cohort has been published [32]. Participants aged 40 to 69 were recruited in two phases (Phase A in 2009–2010 and Phase B in 2013–2014) from the metropolitan areas of Montréal, Québec City, Sherbrooke, Saguenay, Trois-Rivières, and Gatineau in Québec, Canada. Participants completed in-person clinic visits to collect biospecimens (e.g., serum) and physical measures, as well as questionnaires to collect sociodemographic and health history information. The current descriptive analysis includes a sub-sample of female participants who: (1) reported being premenopausal at baseline (Phase A or Phase B), (2) provided a serum sample at baseline, and (3) participated in a 2017 follow-up study of the cohort (in order to facilitate future analyses on prospective associations with health outcomes in this cohort). 2,775 participants met these criteria and were included in the current descriptive analysis. The CARTaGENE study was approved by the research ethics boards of the Université de Montréal and the Centre Hospitalier Universitaire Sainte-Justine (CHU Ste-Justine). The current project was approved by the Health Canada/Public Health Agency of Canada Research Ethics Board. All participants provided informed consent for participation and for the future use of their data and biosamples in research projects.

### Serum collection, laboratory analysis, and quality control

Serum samples were collected in serum separator tubes, transferred to 0.5 mL High Security Sperm straws (Cryo Bio Systems, L’Aigle, France), and stored at -176 °C. Analysis of 40 PFAS was completed between 2021 and 2024 using EPA method 1633 by AXYS Analytical Services Ltd. (MLA-110; Sidney, BC, Canada). The laboratory is ISO 17025 certified by the Canadian Association for Laboratory Accreditation. This included 11 perfluoroalkyl carboxylates, 8 perfluoroalkyl sulfonates, 6 fluorotelomers (3 carboxylates and 3 sulfonates), 7 perfluorooctane sulfonamide-based precursors, 5 per- and polyfluoroether carboxylates including two PFOA replacement compounds (HFPO-DA [GenX] and ADONA), 2 chlorinated polyfluorinated ether sulfonates, and 1 polyfluoroether sulfonate (Supplemental Table 1). Definitions of PFAS have evolved over time and they can be classified in many ways (i.e., according to functional group, chain length, polymers vs. non-polymers, etc.) [1, 33]. In the current paper, we use the term “legacy” when referring to PFAS which have been subject to regulations around the world over the past several decades (e.g., perfluorooctane sulfonate (PFOS), perfluorooctanoic acid (PFOA), perfluorohexane sulfonate (PFHxS), and long-chain perfluoroalkyl carboxylic acids) [18]. We use the term “alternative” to describe PFAS that have either replaced, or may be used in place of, the legacy compounds (e.g., perfluorobutanoic acid (PFBA), ADONA, HFPO-DA, and 9Cl-PF3ONS) [17]. When referring to PFAS compounds that ultimately degrade into the legacy PFAS as their terminal moiety (e.g., the sulfonamide-based precursors MeFOSAA and PFOSA) we use the term “precursor” [19]. This is an imperfect classification scheme, leaving gaps for some PFAS and overlap between others, but it is useful in interpreting human biomonitoring data for PFAS within the context of population-level trends in exposure [16] as well as existing regulatory frameworks [4].

Serum samples were extracted and cleaned up by solid phase extraction and extracts were analyzed by ultra-performance liquid chromatography on a reversed phase C18 column using a solvent gradient. The column was coupled to a triple quadrupole mass spectrometer operated in the multiple reaction monitoring mode, using negative electrospray ionization. After initial calibration, a mid-level calibration was analyzed after every 12 h. Final sample concentrations were determined by SGS AXYS using isotope dilution. Concentrations were corrected for percent recovery of isotopically labelled surrogates within each sample. Limits of detection (LOD) were assigned by determining the concentration at which the peak area was equivalent to 3.0 times the estimated chromatographic noise height. The median and inter-quartile range of LODs across 139 batches (≈ 20 samples/batch) are shown in Supplemental Table 2. PFAS concentrations were blank corrected by subtraction using a de-ionized water blank included in each batch and sample non-detect values were replaced with one-half of the batch-specific blank LOD. Non-positive blank-corrected values were treated as non-detects.

Several quality control samples were analyzed to evaluate method performance. In each batch, de-ionized blank water samples were spiked with 24 isotopically labelled surrogates to provide an indication of method performance within the blank samples. Percent recovery in the blank samples was within the acceptable range (70–130%) for 22/24 surrogates, with the exception of D_9_-N-EtFOSE and ^13^C_2_-4:2 FTS which each had a median percent recovery of 132% across 139 batches (Supplemental Table 3). Each batch also contained two reference serum samples spiked with 40 native PFAS at low and mid-level concentrations (Supplemental Table 4). Percent recovery was within the acceptable range (70–130%) for the majority of PFAS, with the exception of NFDHA and 3:3 FTCA. For NFDHA, 37/139 low concentration reference samples were above 130% recovery and the coefficient of variation of percent recovery across batches was high (54.8% and 56.8% for low and high spike samples, respectively). The percent recovery for 3:3 FTCA was < 70% for all batches for both low and high spike concentrations. Due to the need for cautious interpretation of NFDHA and 3:3 FTCA results, we present only the detection frequencies for these two PFAS.

We conducted additional quality control analyses to ensure that sample concentrations would not be influenced by the storage material (high security sperm straws) (Supplemental Table 5). First, PFAS concentrations were measured in extracts from three blank straws following a 50% formic acid rinse as well as three additional blank straws following a methanol rinse. No PFAS were detected in the extracts from any of the blank straws. Second, to ensure adequate recovery, three blank straws were filled with a reference plasma sample spiked with 40 native PFAS and the percent recovery after rinsing with methanol was calculated. For most PFAS, the average percent recovery was acceptable, ranging from 82 to 113%, with exception of 3:3 FTCA (17.4%) and 5:3 FTCA (60.3%). Finally, after the initial rinse of the straws containing spiked plasma samples, a second elution was performed to determine if any PFAS remained in the straw following extraction; none of the samples had detectable concentrations of PFAS following the second methanol rinse, indicating minimal adsorption to the interior straw surface.

### Covariate data collection

Participants completed detailed questionnaires to provide sociodemographic, lifestyle, and obstetrical history information. Participants reported their age, level of education (high school or less, college diploma, university degree, graduate studies), and cigarette smoking status (never smoker, former smoker, daily/occasional smoker). All participants reported their country of birth and those born outside of Canada also reported their year of immigration or age at immigration. Using this information we derived participants’ time spent living in Canada (< 15 years vs. ≥ 15 years). In phase A, body mass index (BMI) was calculated using measured height and weight and in phase B, BMI was calculated using self reported height and weight. Physical activity was assessed using the International Physical Activity Questionnaire (IPAQ) and categorized as low, moderate or high [34]. Household income was categorized according to the low-income cut-off established by Statistics Canada based on participants’ self-reported total annual household income, number of individuals in the household, population size of the metropolitan area, and year [35]. These low-income thresholds represent the income levels at which households would be expected to spend ≥ 20% more than the average household on food, shelter and clothing [36]. Self-reported race and ethnicity was derived from the following options: White/European, Black African, Arab, Latino, Southeast Asian, East Asian, West Asian, South Asian, Jewish, Caribbean, Mixed Ethnicity, and Other. In this analysis, participants who exclusively identified their race or ethnicity as “White” were coded as being White and participants who identified otherwise (including mixed race/ethnicity) were coded as “Other”. Parity (0, 1, 2, and ≥ 3) and time since last pregnancy (< 10 years, ≥ 10–20 years, ≥ 20 years, never pregnant) were derived using participant’s self-reported obstetrical information. All participants reported if they had ever breastfed, but only those who participated in Phase A reported their lifetime duration of breastfeeding (0–5 months, 6–11 months, ≥ 12 months).

### Statistical analysis

Statistical analyses were performed in SAS Enterprise Guide 7.1 (SAS institute, Cary, NC.) and data visualization was done using R v. 3.6.2 (R Core Team, 2020. R Foundation for Statistical Computing, Vienna, Austria). For PFAS detected in > 60% of samples, we calculated geometric means and 95% confidence intervals (95% CI) as well as Spearman correlation coefficients. We also analyzed the arithmetic sum of the concentrations of 7 PFAS (Σ7PFAS: PFOS, PFNA, PFOA, PFHxS, PFDA, PFUnA, MeFOSAA), as recommended in the clinical testing guidance proposed by the National Academics of Sciences, Engineering, and Medicine [9]. We used general linear models with log-transformed PFAS concentrations to calculate the geometric mean (95% CI) concentrations within strata of sociodemographic, lifestyle, and reproductive history characteristics (i.e., parity, time since last pregnancy, and breastfeeding history) and used ANOVA to test for differences between group-specific geometric means. When the overall group effect was statistically significant (*p* < 0.05), we conducted pairwise comparisons using the Bonferroni method. We also estimated the effect of age, both continuously and categorically, across categories of parity. Continuous regression coefficients for age were back-transformed to represent percent differences in PFAS concentrations using the formula (*e*^β^-1)*100. We considered examining joint relationships between age and time since last pregnancy, but these factors were highly correlated (spearman’s rho = 0.7) and their interactions could not be modelled because of collinearity.

## Results

Participants were mostly White (91%), born in Canada (89%), highly educated (87% with post-secondary education), and 86% reported a household income above Statistics Canada’s low-income cut-off (Table 1). The majority of participants had 2 or more children and 61% reported ever having breastfed. Among 628 nulliparous participants, 441 reported having never been pregnant, while 183 participants reported having been pregnant but without a birth after 20 weeks gestation, and 4 participants did not report their time since last pregnancy.

**Table 1:** Sociodemographic, lifestyle, and reproductive history characteristics of premenopausal adult female participants in the CARTaGENE study (2009–2014; *n* = 2775)

	*n*	%
Phase		
A (2009–2010)	1616	58
B (2013–2014)	1159	42
Age (years)		
< 45	814	29
45–49	1121	40
≥ 50	840	30
Country of birth		
Canada	2475	89
Outside of Canada	300	11
Time living in Canada (years)		
< 15	116	4
≥ 15	2657	96
Missing	2	0.1
Race and ethnicity		
White	2523	91
Other	232	8
Missing	20	0.7
Body mass index (kg/m^2^)		
< 25	1251	45
25–29	765	28
≥ 30	514	19
Missing	245	9
Smoking status		
Never smoker	1434	52
Former smoker	1002	36
Daily/Occasional smoker	326	12
Missing	13	0.5
Parity		
0	628	23
1	484	17
2	1096	40
≥ 3	567	20
Time since last pregnancy (years)		
< 10	436	16
≥ 10–20	1218	44
≥ 20	674	24
Never Pregnant	441	16
Missing	6	0.2
History of breastfeeding		
Yes	1696	61
No	883	32
Missing	196	7
Lifetime duration of breastfeeding (months)^1^		
0–5	281	17
6–11	272	17
≥ 12	378	23
NA	557	35
Missing	128	8
Level of education		
High school or less	340	12
College diploma	856	31
University degree	1099	40
Graduate studies	470	17
Missing	10	0.4
Below Canadian low income cutoff^2^		
Yes	162	6
No	2396	86
Missing	217	8
Physical Activity (IPAQ)		
Low	553	20
Moderate	1114	40
High	983	35
Missing	125	5

*IPAQ* International Physical Activity Questionnaire. Percentages may not add to 100% due to rounding

^1^ Includes participants from CARTaGENE phase A only. NA includes individuals who did not report a history of pregnancy or breastfeeding and were not offered the question in Phase A

^2^ Household income was categorized according to the low-income cut-off established by Statistics Canada based on participants’ self-reported total annual household income, number of individuals in the household, population size of the metropolitan area, and year

Eight out of 40 PFAS were detected in > 60% of samples, four of which were detected in > 99% of samples (Table 2). The geometric means of these PFAS ranged from 0.03 µg/L (MeFOSAA and PFHpS) to 4.25 µg/L for PFOS and 8.00 µg/L for the Σ7PFAS. Spearman correlations between these PFAS were highest among PFOS, PFNA, PFOA, PFHxS, PFDA, PFUnA, and PFHpS (up to 0.68 between PFOA and PFOS); correlations of these 7 PFAS were generally lower with MeFOSAA (Supplemental Fig. 1). The 95^th^ percentiles of the remaining 32 PFAS were < 0.5 µg/L, and many were < 0.1 µg/L (Table 2).

**Table 2:** Detection frequencies and descriptive statistics for serum concentrations of per- and polyfluoroalkyl substances (µg/L) measured in adult female participants from the CARTaGENE cohort (2009–2014)

	*n*	Detection frequency	GM (95% CI)	25^th^ percentile	50^th^ percentile	75^th^ percentile	95^th^ percentile
Σ7PFAS	2774	-	8.00 (7.84, 8.16)	5.74	8.16	11.49	17.99
PFOS	2774	100	4.25 (4.16, 4.35)	2.90	4.36	6.48	11.33
PFNA	2774	99.8	0.71 (0.69, 0.72)	0.53	0.73	0.94	1.46
PFOA	2774	99.6	1.55 (1.52, 1.58)	1.15	1.58	2.13	3.51
PFHxS	2774	99.6	0.57 (0.55, 0.59)	0.35	0.57	0.92	2.17
PFDA	2774	89.5	0.24 (0.23, 0.24)	0.18	0.27	0.38	0.62
PFUnA	2774	84.2	0.18 (0.18, 0.19)	0.12	0.22	0.34	0.61
PFHpS	2774	78.6	0.03 (0.03, 0.04)	0.02	0.06	0.12	0.23
MeFOSAA	2774	68.0	0.03 (0.02, 0.03)	-	0.03	0.09	0.29
PFOSA	2774	52.5	-	-	< 0.01	0.01	0.03
8:2 FTS	2774	46.0	-	-	-	0.03	0.09
PFBA	2774	39.8	-	-	-	0.10	0.20
PFHpA	2774	35.7	-	-	-	0.08	0.18
6:2 FTS	2774	35.3	-	-	-	0.06	0.23
4:2 FTS	2775	34.7	-	-	-	0.01	0.03
MeFOSA	2774	30.7	-	-	-	0.01	0.04
EtFOSA	2774	30.5	-	-	-	0.01	0.05
HFPO-DA	2775	30.1	-	-	-	0.01	0.03
PFTrDA	2775	28.8	-	-	-	0.09	0.28
EtFOSE	2774	28.8	-	-	-	0.01	0.04
PFBS	2774	26.2	-	-	-	0.01	0.06
PFPeA	2775	25.1	-	-	-	0.05	0.12
PFDoA	2773	24.5	-	-	-	-	0.14
MeFOSE	2774	24.4	-	-	-	-	0.07
PFMBA	2774	23.4	-	-	-	-	0.01
PFHxA	2774	23.0	-	-	-	-	0.12
PFTeDA	2774	22.4	-	-	-	-	0.21
9Cl-PF3ONS	2774	22.1	-	-	-	-	0.02
PFPeS	2774	21.2	-	-	-	-	0.03
EtFOSAA	2774	20.6	-	-	-	-	0.05
PFDS	2774	20.0	-	-	-	-	0.03
7:3 FTCA	2774	19.4	-	-	-	-	0.45
ADONA	2774	12.5	-	-	-	-	< 0.01
5:3 FTCA	2774	11.1	-	-	-	-	0.20
PFMPA	2773	9.4	-	-	-	-	0.02
PFNS	2774	5.8	-	-	-	-	0.01
PFEESA	2774	4.1	-	-	-	-	-
PFDoS	2774	3.6	-	-	-	-	-
11Cl-PF3OUdS	2774	3.4	-	-	-	-	-
NFDHA^1^	2774	2.5	-	-	-	-	-
3:3 FTCA^1^	2773	0.4	-	-	-	-	-

*Σ7PFAS* Sum of PFOS, PFNA, PFOA, PFHxS, PFDA, PFUnA, and MeFOSAA as recommended by the National Academies of Sciences, Engineering, and Medicine’s Guidance on PFAS Exposure, Testing, and Clinical Follow-Up

^1^ Interpret with caution due to low percent recovery (3:3 FTCA) and high coefficients of variation (NFDHA)

**Fig. 1:**
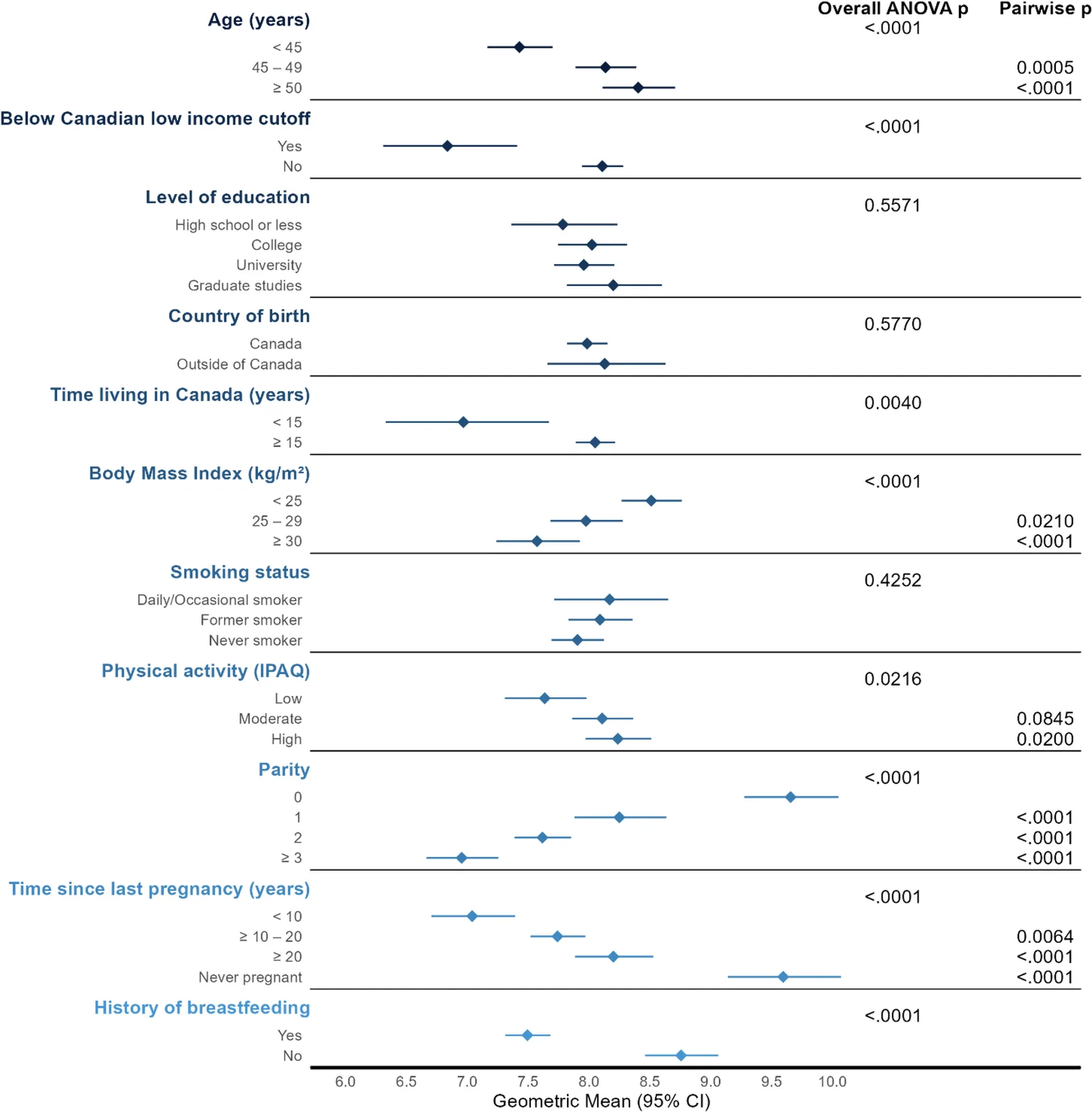
Geometric mean (95% CI) serum concentrations of the sum of 7 PFAS (PFOS, PFNA, PFOA, PFHxS, PFDA, PFUnA, MeFOSAA) (µg/L) across sociodemographic, lifestyle, and reproductive history characteristics among adult females in the CARTaGENE cohort (2009–2014)

**Fig. 2:**
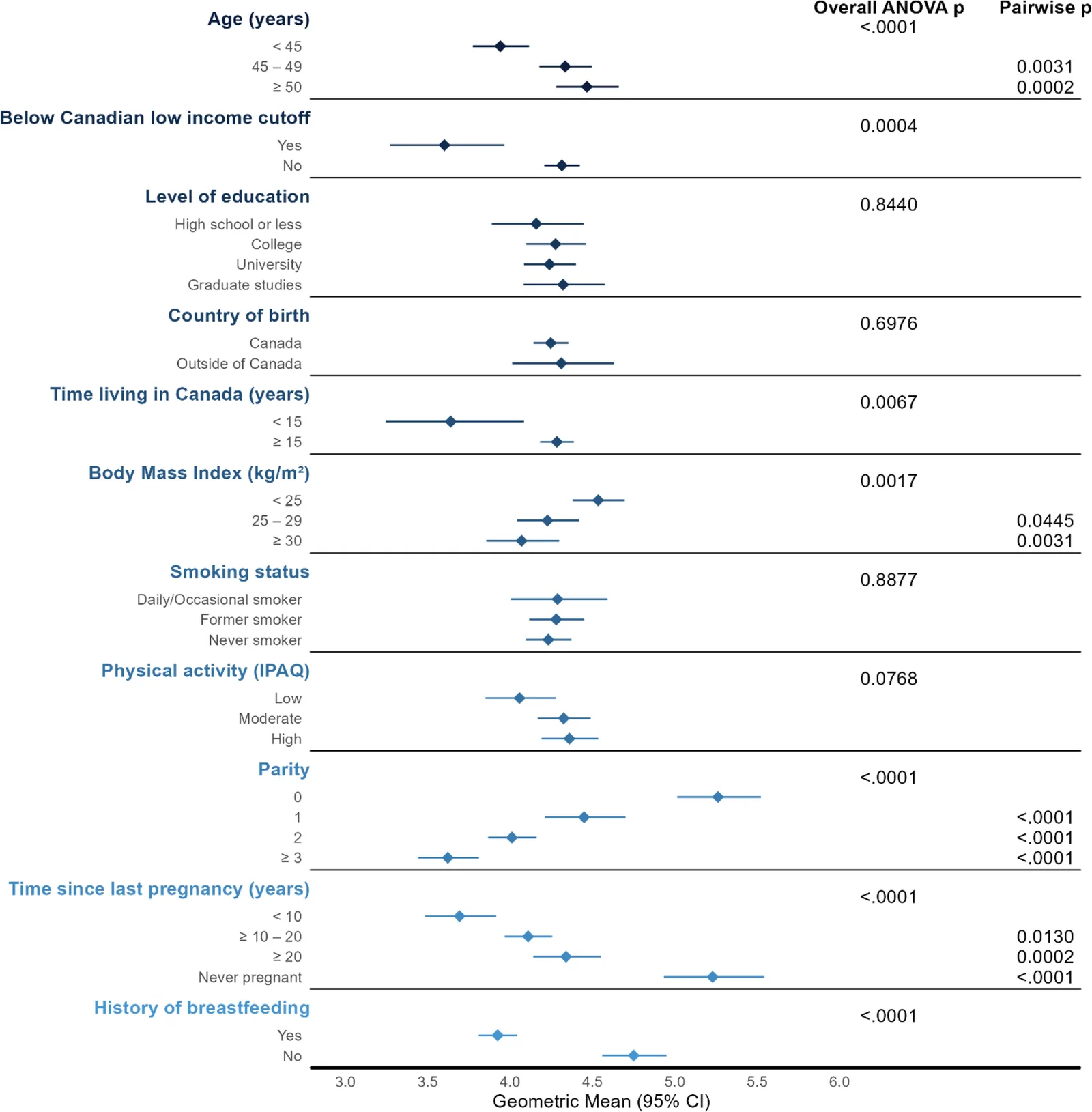
Geometric mean (95% CI) serum concentrations of PFOS (µg/L) across sociodemographic, lifestyle, and reproductive history characteristics among adult females in the CARTaGENE cohort (2009–2014)

**Fig. 3:**
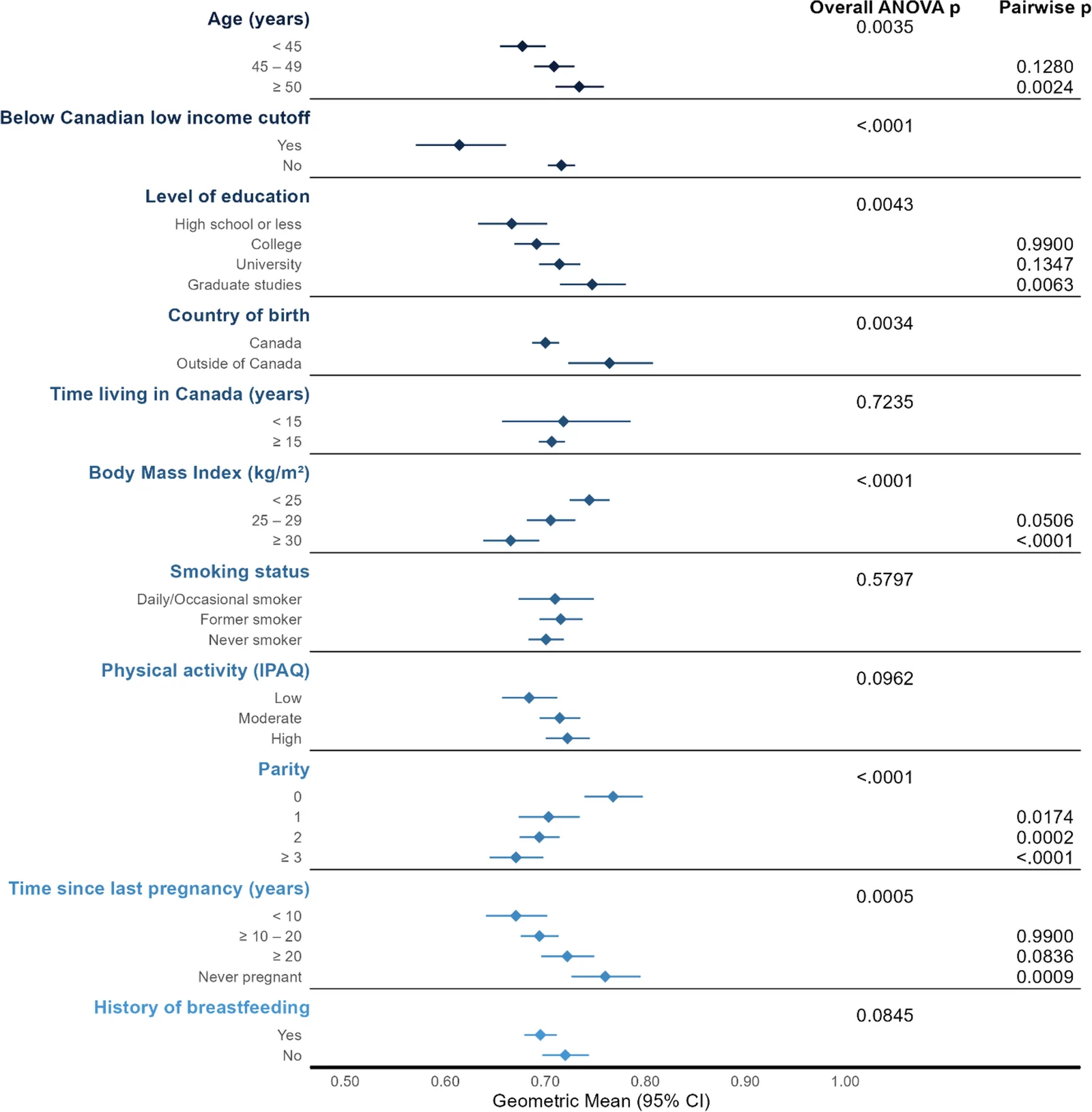
Geometric mean (95% CI) serum concentrations of PFNA (µg/L) across sociodemographic, lifestyle, and reproductive history characteristics among adult females in the CARTaGENE cohort (2009–2014)

**Fig. 4:**
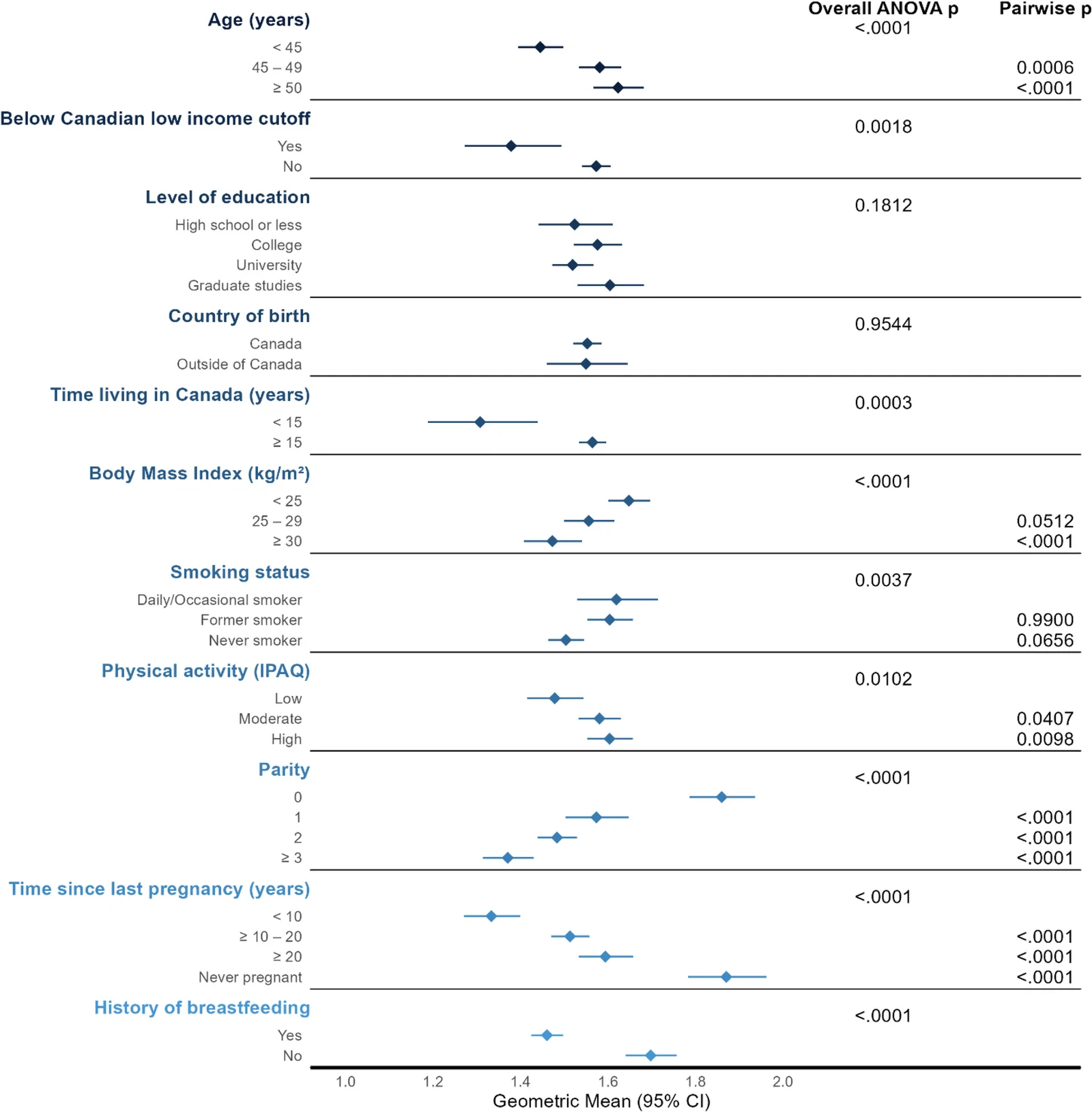
Geometric mean (95% CI) serum concentrations of PFOA (µg/L) across sociodemographic, lifestyle, and reproductive history characteristics among adult females in the CARTaGENE cohort (2009–2014)

**Fig. 5:**
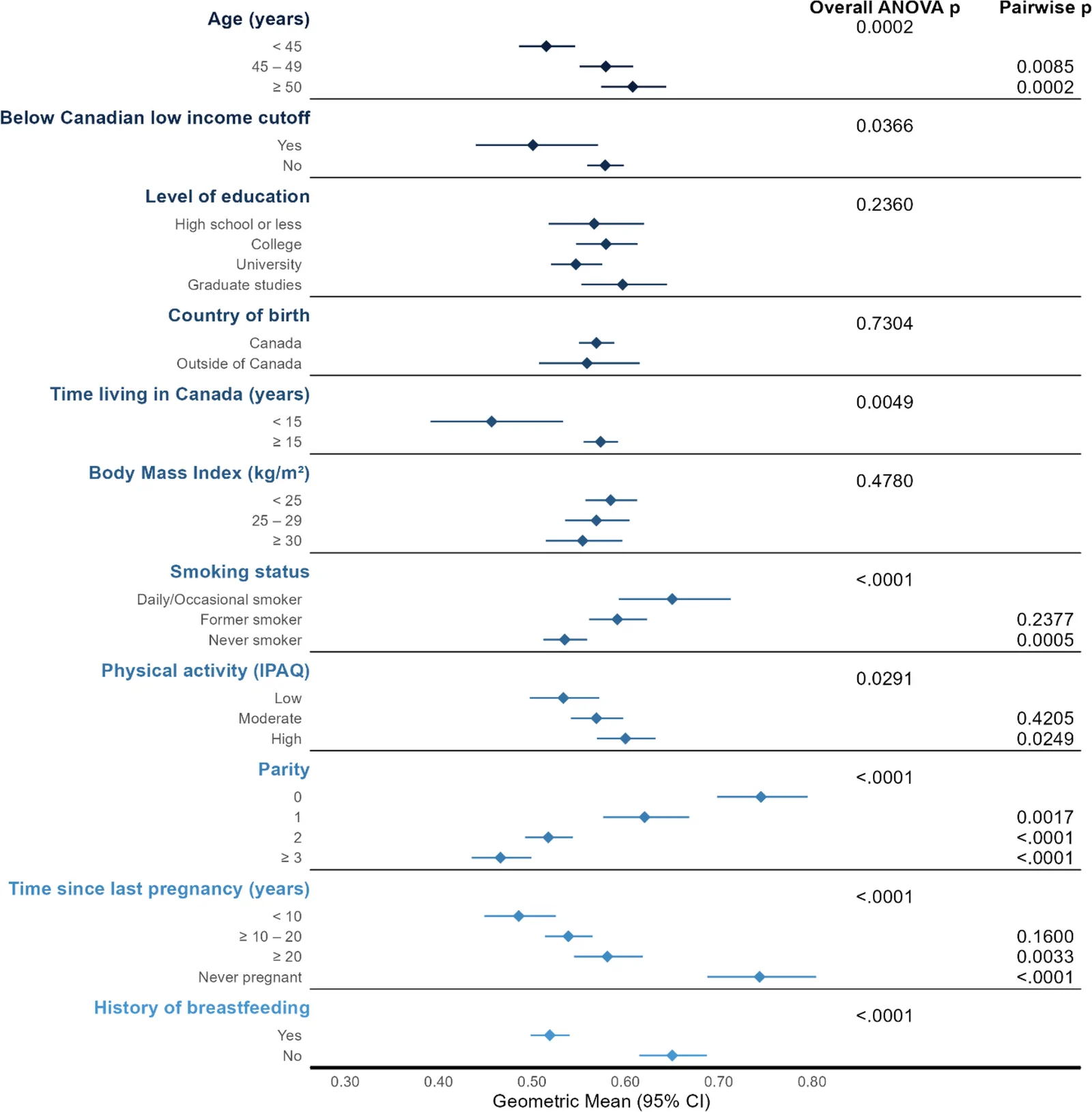
Geometric mean (95% CI) serum concentrations of PFHxS (µg/L) across sociodemographic, lifestyle, and reproductive history characteristics among adult females in the CARTaGENE cohort (2009–2014)

**Fig. 6:**
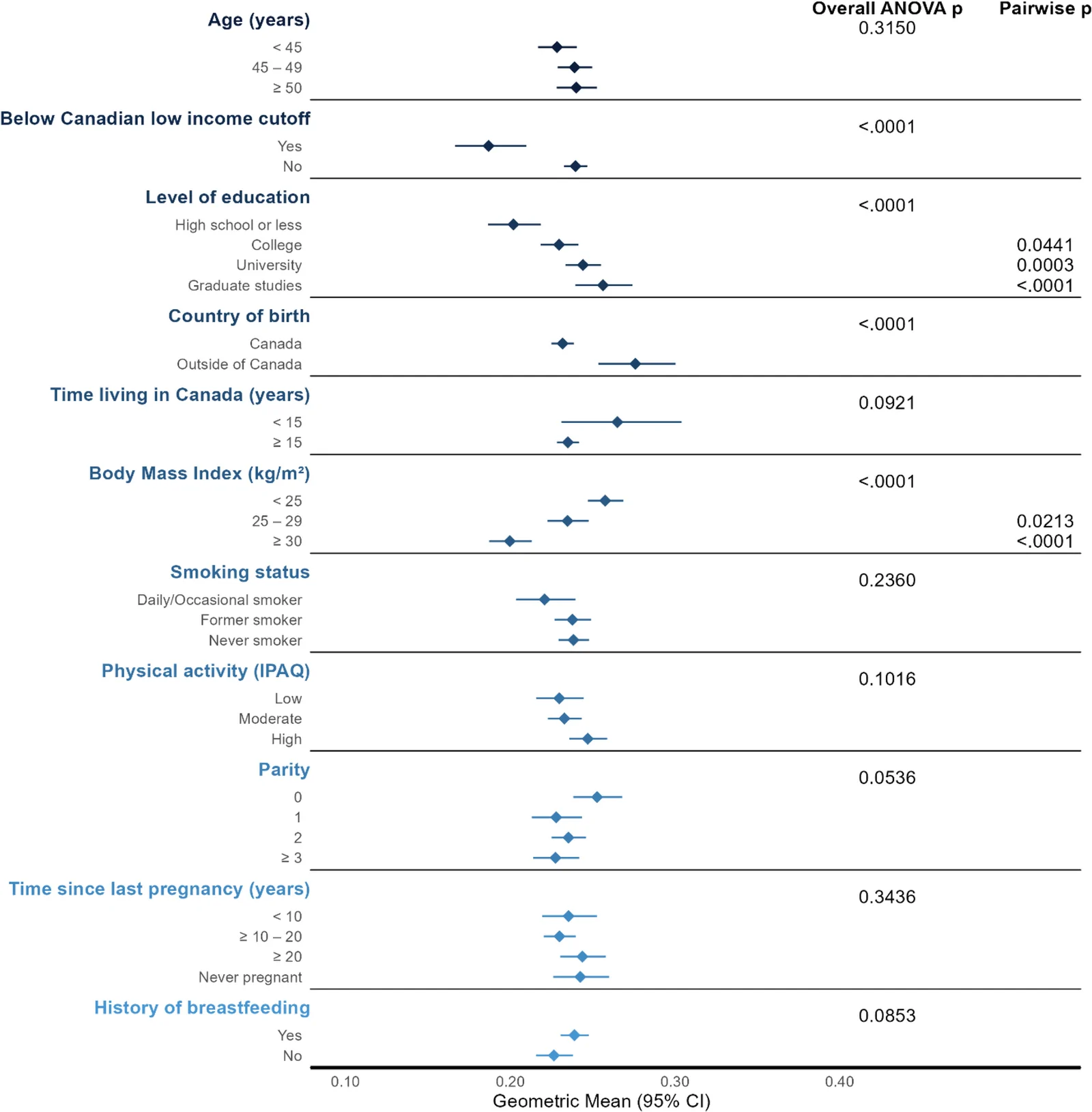
Geometric mean (95% CI) serum concentrations of PFDA (µg/L) across sociodemographic, lifestyle, and reproductive history characteristics among adult females in the CARTaGENE cohort (2009–2014)

**Fig. 7:**
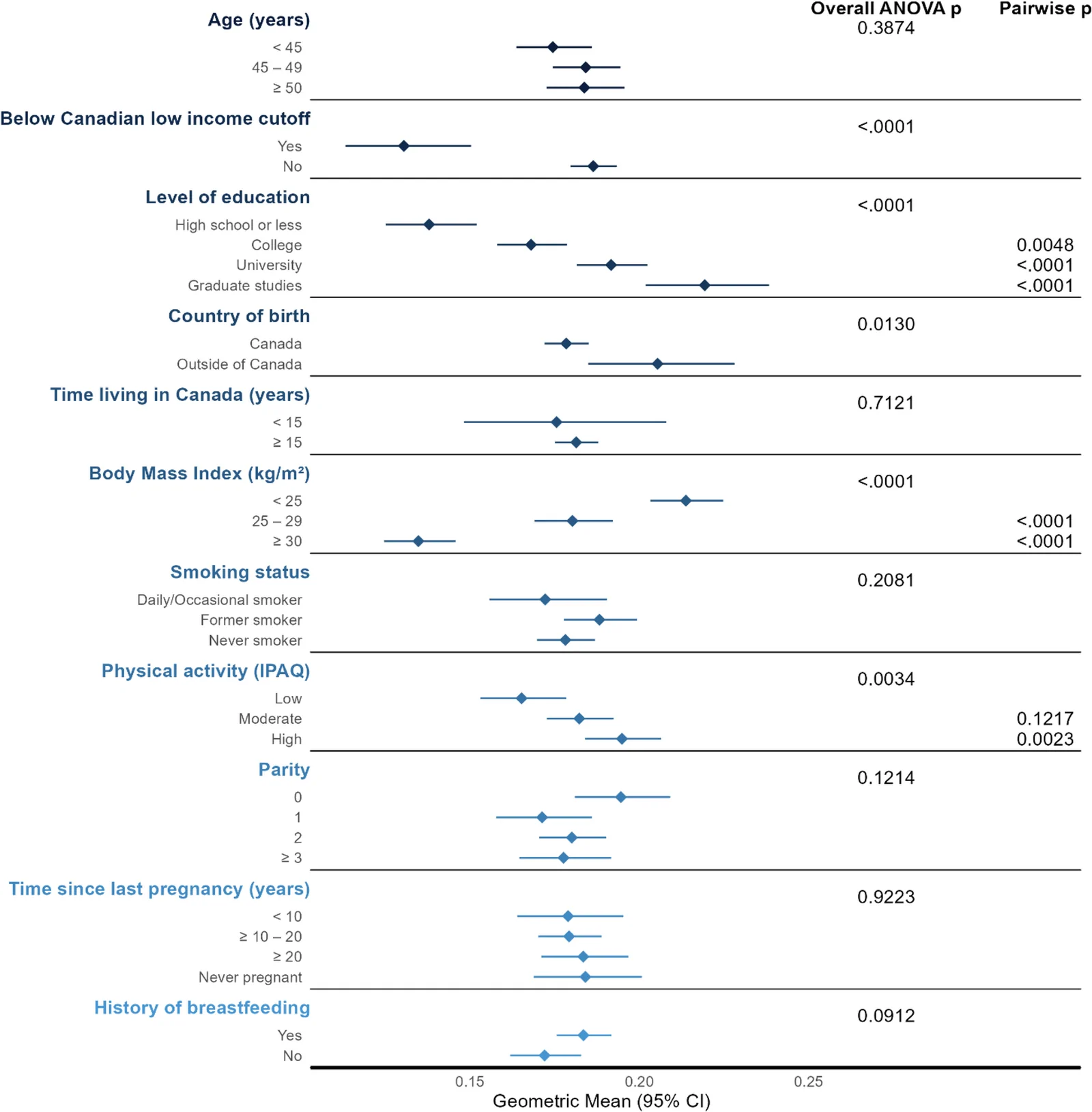
Geometric mean (95% CI) serum concentrations of PFUnA (µg/L) across sociodemographic, lifestyle, and reproductive history characteristics among adult females in the CARTaGENE cohort (2009–2014)

Geometric mean serum concentrations of the 8 PFAS with > 60% detection and the Σ7PFAS across categories of sociodemographic, lifestyle, and reproductive history characteristics are presented in Figs. 1, 2, 3, 4, 5, 6 and 7 and Supplemental Tables 6–10. Concentrations of PFHpS and MeFOSAA (Supplemental Table 10) were similar across all characteristics examined, with some small, yet statistically significant, differences ranging from 0.02 to 0.05 µg/L, reflecting the low level of exposure to these PFAS in this study. Serum concentrations of the remaining 6 PFAS and the Σ7PFAS were considerably lower among those enrolled in CARTaGENE phase B (2013–2014) vs. phase A (2009–2010) (Supplemental Tables 6–9). Aside from recruitment phase, the largest differences in serum PFAS concentrations were observed across reproductive history characteristics, with concentrations differing by up to 38% when comparing the highest vs. lowest categories of these factors. Concentrations of PFOS, PFOA, PFNA, PFHxS, and the Σ7PFAS were monotonically lower with each additional child birthed (from 0 to ≥ 3) and were higher with increasing time since last pregnancy (Figs. 1, 2, 3, 4 and 5). With the exception of PFNA, these PFAS were also lower among participants with a history of breastfeeding. In contrast, serum concentrations of PFDA (Fig. 6) and PFUnA (Fig. 7) were similar across reproductive history characteristics.

Serum concentrations of most PFAS examined (except for PFHpS and MeFOSAA) were lower among those with a household income below the low-income cut-off, while concentrations were higher with increasing level of education for PFNA, PFDA, and PFUnA. Concentrations of PFOS, PFOA, and PFHxS and the Σ7PFAS were lower among participants with < 15 years lived in Canada (but similar for country of birth), while concentrations of PFNA, PFDA, and PFUnA were lower among those born in Canada (but similar across time spent in Canada). Participants with obesity had lower serum concentrations of most PFAS (except PFHxS) compared to those with under/normal weight. Participants reporting low levels of physical activity had slightly lower serum concentrations of PFOA, PFHxS, PFUnA, and the Σ7PFAS. Current smokers had higher serum PFHxS concentrations than never smokers; this trend was not observed for any other PFAS. No differences were observed for race/ethnicity for any of the PFAS (results in Additional file 1).

**Fig. 8:**
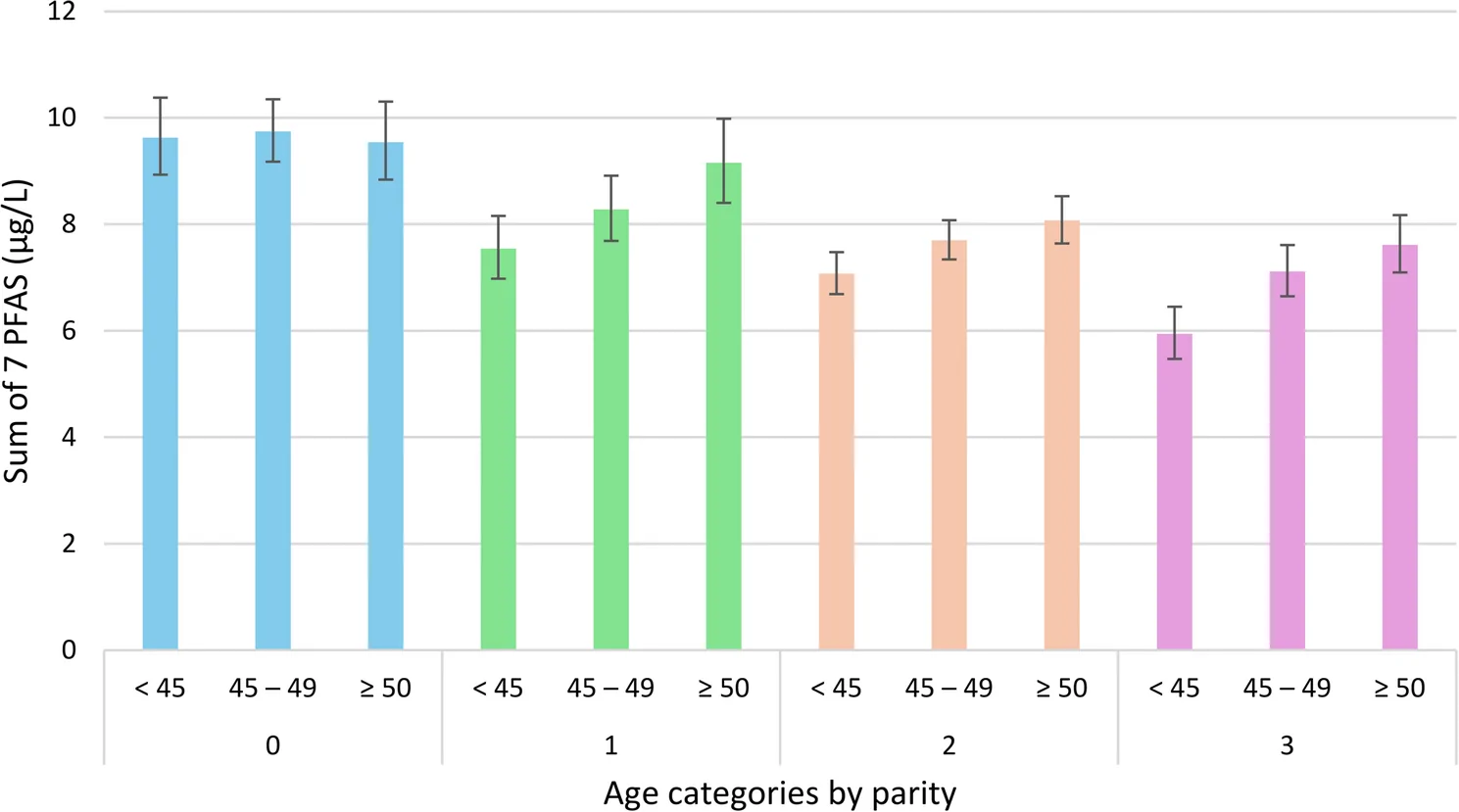
Geometric mean (95% CI) sum of 7 PFAS (PFOS, PFNA, PFOA, PFHxS, PFDA, PFUnA, MeFOSAA) (µg/L) serum concentrations by age category across parity groups among adult females in the CARTaGENE cohort (2009–2014)

Older individuals had higher concentrations of PFOS, PFOA, PFNA, PFHxS and the Σ7PFAS (Figs. 1, 2, 3, 4 and 5), but not PFDA or PFUnA (Figs. 6 and 7). However, when examined across categories of parity, we found that concentrations of PFOS, PFOA, PFNA, PFHxS, and Σ7PFAS were higher with increasing age among parous and multiparous women, but no associations were observed for age among nulliparous women (Supplemental Table 11). For example, each 1-year increase in age was associated with 1.7% (95%CI: 0.6, 2.7), 1.4% (95%CI: 0.7, 2.1), and 2.4% (95%CI: 1.5, 3.4) higher Σ7PFAS concentrations among those with 1, 2, or 3 prior pregnancies, respectively. Geometric means (95%CI) for the Σ7PFAS by age category across parity groups are shown in Fig. 8 to illustrate the effect of both parity and age on serum PFAS concentrations; results for the individual PFAS are shown in Supplemental Figs. 3–10. Similar to the full sample results, age was not associated with PFDA or PFUnA concentrations across all categories of parity, while small, yet statistically significant, associations were observed for MeFOSAA and PFHpS. Age was similarly distributed across categories of parity, with median (interquartile range) ages of 48 (45–50), 47 (44–51), 48 (44–51), and 48 (45–52) years for individuals with 0, 1, 2, and 3 prior pregnancies, respectively.

## Discussion

We report human biomonitoring data for legacy, alternative, and precursor PFAS among a large sample of premenopausal participants from the Québec-based CARTaGENE cohort (2009–2014). Eight PFAS – six of which were legacy or long-chain PFAS – were detected in > 60% of samples. Serum concentrations of these PFAS were generally lower among participants who were multiparous, had a more recent pregnancy, and a history of breastfeeding. Concentrations of these PFAS were also lower among those with a household income below the Canadian low-income cut-off. Several alternative and precursor PFAS were detected in participants in this population (including in > 60% of samples for PFHpS and MeFOSAA), though serum concentrations of these PFAS were relatively low (i.e., 95^th^ percentiles < 0.5 µg/L).

The detection frequencies and concentrations of PFAS in the present study are similar to available data for nine PFAS from the 2009–2010 Canadian Health Measures Survey (CHMS) for females aged 40–59 [37] (Supplemental Fig. 2), with some exceptions. For PFOS and PFOA, concentrations among participants recruited in CARTaGENE phase A (2009–2010) were more similar to nationally representative data collected during the same time frame; the lower concentrations among Phase B (2023–2014) participants is consistent with established trends of declining PFOS and PFOA exposure in Canada [16]. Detection frequencies of PFBA, PFHxA, and PFBS were higher in the current study than the CHMS likely because of the more sensitive method used; the 95^th^ percentiles for these PFAS in the current study were less than or similar to the LODs used in the CHMS.

For alternative and precursor PFAS, the low serum concentrations observed in our study are consistent with the limited existing Canadian biomonitoring data from the 2010–2011 CHILD pregnancy cohort [38] and a 2018–2021 follow-up study of non-pregnant participants from the MIREC pregnancy cohort (MIREC-ENDO) [21]. In the CHILD cohort, median plasma concentrations of PFHxA, PFHpA, PFBS, PFDS, PFOSA, MeFOSE, MeFOSA, EtFOSE, and EtFOSA were below the study-specific limits of quantification (LOQs). The LOQs in CHILD were similar to the 95^th^ percentiles in our study. In the MIREC-ENDO study, 95^th^ percentile serum concentrations were similarly < 0.5 µg/L for nearly all alternative and precursor PFAS (with exception of 7:3 FTCA, which was 1.5 µg/L). Differences in the 95^th^ percentiles between the current and MIREC-ENDO studies were nearly all < 0.2 µg/L. The higher detection frequencies observed for several alternative and precursor PFAS in the MIREC-ENDO study can be explained by the method of handling non-positive blank-corrected values. In MIREC-ENDO, these values were treated as being detected, while the current study used an alternative approach and treated these values as non-detects. Collectively, these studies suggest that Canadians’ exposure to alternative and precursor PFAS may have been relatively low between 2009 and 2021, and that exposure appears to have remained relatively unchanged over this time period. However, these studies included only female participants during middle adulthood from predominately urban/suburban settings, and may not be generalizable to the larger Canadian population. Biomonitoring data for alternative and precursor PFAS in more diverse and representative populations are needed to confirm these findings. Moreover, emerging data suggests that some of alternative or precursor PFAS, particularly those with shorter carbon chains, may have shorter biological half-lives on the order of days to months [39, 40], compared to years for the legacy PFAS [41, 42]. This difference in bioaccumulation potential could help to explain the relatively low concentrations of alternative or precursor PFAS observed in these studies.

Consistent with current evidence that pregnancy and breastfeeding act as excretion pathways for PFAS [27, 29, 43], we showed that greater parity, shorter time since last pregnancy, and breastfeeding history were associated with significantly lower concentrations of most legacy PFAS. Concentrations of PFDA and PFUnA did not follow this pattern, which may reflect their limited transfer into breastmilk, as multiple studies have reported low detection frequencies and low concentrations for these PFAS in breastmilk [44–49]. Much of the previous evidence for associations with reproductive history characteristics is derived from pregnancy cohorts [21, 25], which, by design, exclude participants who have never been pregnant. Our descriptive analysis complements these previous investigations by using an older, general-population cohort where we observed that female participants with no history of pregnancy or deliveries beyond 20 weeks (i.e., parity of 0) consistently had the highest concentrations of PFOS, PFOA, PFNA, and PFHxS. While similar patterns of differences were observed for PFHpS and MeFOSAA, the magnitude of these differences were smaller. In addition to its role as an excretion pathway, emerging research suggests that lactation may be compromised by exposure to PFAS [50]. Five out of six studies included in a 2023 systematic review found that individuals with higher PFAS serum concentrations were more likely to discontinue breastfeeding earlier in pregnancy [51]. Breastfeeding is a complex biological and sociocultural behaviour, and while the reasons for cessation of breastfeeding are varied [52], exposure to PFAS may play a role. Participants in CARTaGENE (Phase A only) reported a relatively short amount of time spent breastfeeding compared to other studies, which may explain why we did not observe differences in PFAS concentrations across categories of lifetime duration of breastfeeding. In the Canadian MIREC-ENDO study, serum concentrations of PFOS, PFOA, and PFHxS were lower among those with a lifetime duration of breastfeeding > 4 years vs. ≤ 2 years [21], while in the current study 89% of participants reported breastfeeding for fewer than 2 years. The reasons for this difference may be both geographical and temporal – rates of exclusive breastfeeding in the first 4–6 months are lower in Québec compared to other Canadian provinces [53]. Moreover, despite having increased to 29% in 2020–2021 in Québec, rates were as low as 6% in the late 1990s [54] during the timeframe of many of the pregnancies among CARTaGENE participants. Finally, results from the Danish National Birth Cohort and the Canadian MIREC-ENDO study previously demonstrated that legacy PFAS bioaccumulate in serum during the inter-pregnancy interval [25] and up to 8 years post-partum [21], respectively. Our analysis extends these previous investigations by showing that legacy PFAS continue to bioaccumulate from the post-partum period and up to 20 or more years later. Future studies should consider parity, time since last pregnancy, and breastfeeding history as potential confounders of associations between PFAS and health outcomes that are influenced by these reproductive history characteristics.

We observed a consistent pattern of higher PFAS concentrations with higher socioeconomic status. Serum concentrations of nearly all of the highly detected PFAS were lower among participants with a household income below the Canadian low-income cut-off. Concentrations were similar or slightly higher (e.g., PFNA, PFDA, and PFUnA) with increasing level of education. These findings are consistent with a 2018 meta-analysis of PFAS exposure and socioeconomic status among North American and European studies, which found that each doubling of household income was associated with 10–14% higher serum/plasma concentrations of PFOS, PFOA, PFNA, and PFHxS [55]. This is also consistent with a 2023 systematic review of determinants of PFAS exposure among pregnant women [26], with most identified cohort studies finding that income is a positive predictor of PFOS, PFOA, PFNA, and PFHxS. Our findings are also consistent with three additional studies published since this 2023 systematic review [56–58]. Data from the 2013–2018 US NHANES suggest that concentrations of a mixture of mostly legacy PFAS were monotonically higher with increasing household income [56]. In the 2014–2016 Survey of the Health of Wisconsin (SHOW) Cohort, each doubling of household income above the US federal poverty level was associated with 3–4% higher serum concentrations of PFOS, PFOA, PFHxS, and PFNA [57]. The SHOW cohort included participants with a greater diversity of household income than the current study, with ≈ 40% of participants reporting a household income < USD$50,000. Finally, in the 2009–2010 National Children’s Study [58], household income was a significant predictor of serum PFOS, PFOS, PFNA, PFHxS, and PFDA concentrations in multivariate models adjusting for age, race/ethnicity, education level, household size, home age, and drinking water source. The exact causes of income-related differences in serum PFAS concentrations remain unclear. Possible explanations include differences in intake of PFAS through diet, either through food directly or food contact materials, differences in the types and/or volume of consumer products purchased, or differences in occupational sources of exposure (e.g., office workers exposed to PFAS through carpet and furniture treatments [59, 60]). Collectively, these findings should motivate future research to identify potential differences in sources of exposure among disproportionately exposed populations in Canada.

The effects of parity and age on serum PFAS concentrations are well established [26], but the interaction between these apparently opposing effects is poorly understood. Our results suggest that while these are independent effects, the PFAS reducing effect of parity appears to be stronger than the positive effect of age over approximately 10–20 years during the premenopausal period and at levels of exposure consistent with the general Canadian population. Older multiparous participants had lower levels of several legacy PFAS than younger nulliparous participants, indicating that the effect of parity is not fully countered by the effect of age during the reproductive years. Previous studies have found that long interpregnancy intervals allow for greater potential for age-related PFAS accumulation [61], and have speculated that levels could revert back to those observed in pre-pregnancy during the interpregnancy interval. In addition, using data from the Japanese JECS cohort, Lai et al. (2025) propose that age-related increases in some PFAS could be more important than the reduction attributable to parity [30]. These discrepant findings may be explained by differences in study population and design. These birth cohorts studies included participants that were considerably younger (i.e., 9% of participants were older than 35 years [61], or median age of 31 years [30], vs. 29% of participants younger than 45 years in the current study) and included a lower proportion of multiparous individuals (12% [61] and 22% [30], respectively). In contrast, the current study included participants within 10–15 years of the expected age at menopause with a higher proportion of multiparous individuals (60%), allowing for a more fulsome analysis of these effects across the reproductive years. It is unclear why we did not observe any association between age and PFAS concentrations among nulliparous participants in this study. There is limited comparable evidence for this finding, as nulliparous individuals are often excluded by design (e.g., in birth cohort studies) or not specifically identified (e.g., in general population studies) in research examining age-related effects on PFAS during midlife. Replication of this finding in other populations is warranted.

This study has several strengths, including the large sample size and availability of several sociodemographic, lifestyle, and reproductive history characteristics. Including nulliparous participants and restricting analyses to premenopausal women allowed us to investigate the joint effects of parity and age on PFAS serum concentrations which helps to advance our understanding of changes in PFAS body burden over the life course. The CARTaGENE cohort was designed to be representative of the metropolitan population of Québec at the time of recruitment [32]. While our findings for legacy PFAS are consistent with existing nationally representative data in Canada, the limited data for alternative and precursor PFAS precludes us from determining whether our findings for these PFAS are generalizable to the broader Canadian population. Gaps in biomonitoring data for these PFAS in Canada remain, including among males, younger and older adult females, and individuals living in rural or remote areas, among others. For the alternative and precursor PFAS examined in our study, the only comparable Canadian data from the MIREC-ENDO study were also derived from a sample of adult females in midlife recruited from mostly urban/suburban communities [21]. Additional human biomonitoring data for alternative and precursor PFAS are needed to determine if the low levels of exposure observed in these two studies are consistent with exposure levels in the general Canadian population. In line with the larger CARTaGENE cohort [32], the majority of the participants in this study reported their race/ethnicity as White, which limited our ability to investigate potential differences in exposure levels across racial and ethnic sub-groups. Finally, the presence of a chemical in blood does not necessarily mean that this chemical is associated with an adverse health effect. While the health effects of exposure to some legacy PFAS are well established [4, 9–12], further evidence is needed to determine the potential health impacts of exposure to the alternative and precursor PFAS measured in our study, especially at lower levels typically experienced by the general population.

## Conclusion

We report biomonitoring data for legacy, alternative, and precursor PFAS from a large sample of premenopausal Canadians from the Québec-based CARTaGENE cohort. Our results extend previous findings demonstrating that pregnancy and breastfeeding are excretion pathways for legacy PFAS and suggest that the PFAS reducing effect of parity appears to be stronger than the opposing effect of age. We also showed that exposure to some PFAS was lower amongst economically disadvantaged populations. Finally, our results suggest that low levels of exposure to alternative and precursor PFAS may have been widespread from 2009 to 2014, though additional biomonitoring data in more diverse and representative populations are required to confirm this finding.

## Supplementary Information

Supplementary Material 1.

## Data Availability

The datasets generated and analysed for the current study are not publicly available to respect participants’ privacy, but individuals may apply to access the data through the CARTaGENE Biobank (https://cartagene.qc.ca/en/).
