# Supplementary material for: Serum concentrations of legacy, alternative, and precursor per- and polyfluoroalkyl substances: a descriptive analysis of premenopausal participants in the Canadian CARTaGENE cohort
Source: Environ Health. 2026 Aug 19;25:85. doi: 10.1186/s12940-026-01326-3 (PMC13761184; doi:10.1186/s12940-026-01326-3)
Supplement: Supplementary file 1 [file 12940_2026_1326_MOESM1_ESM.docx]

**Supplemental information**

**Serum concentrations of legacy, alternative, and precursor per- and polyfluoroalkyl substances: a descriptive analysis of premenopausal adult female participants in the CARTaGENE study**

Michael M. Borghese^a^, Rebecca Lelievre^a^, Sara Packull-McCormick^a^, Jillian Ashley-Martin^a^, Maria P. Velez^b^, Nolwenn Noisel^c^, Mengting Xu^d^, Jennifer E Bruin^e^, Tyler Pollock^a,^ Annie St-Amand^a^

^a^ Environmental Health Science and Research Bureau, Health Canada, Ottawa, Canada

^b^ Department of Obstetrics and Gynecology, Research Institute McGill University Health Centre, McGill University, Montreal, Canada

^c^ Département de santé environnementale et santé au travail, Université de Montréal, Montréal, Canada

^d^ CARTaGENE, Sainte-Justine University Hospital Research Center, Montreal, Canada

^e^ Department of Biology and Institute of Biochemistry, Carleton University, Ottawa, Canada

Corresponding author:

Michael Borghese, PhD

[Michael.borghese@hc-sc.gc.ca](mailto:Michael.borghese@hc-sc.gc.ca)

Environmental Health Science and Research Bureau, Health Canada, Ottawa, Canada

Table of Contents

[**Supplemental Table 1 –** Abbreviations and full names for the 40 PFAS measured in the CARTaGENE study	4](#_Toc229747173)

[**Supplemental Table 2** – Median and interquartile range of limits of detection (µg/L) for batch-specific blank samples (139 batches)	6](#_Toc229747174)

[**Supplemental Table 3** – Percent recovery of spiked surrogate standards in blank samples from 139 batches in the CARTaGENE study	8](#_Toc229747175)

[**Supplemental Table 4** – Percent recovery of native per- and polyfluoroalkyl substances from two spiked reference serum samples from 139 batches in CARTaGENE study	9](#_Toc229747176)

[**Supplemental Table 5** – PFAS concentrations in clean straws following rinses using 50% formic acid and basic methanol. Results represent the average concentration of analytes in three batches of straws for each type of rinse.	11](#_Toc229747177)

[**Supplemental Table** 6 – Comparison of serum concentrations of the sum of 7 PFAS (PFOS, PFNA, PFOA, PFHxS, PFDA, PFUnA, MeFOSAA) (µg/L) across sociodemographic, lifestyle, and reproductive history characteristics among adult females in the CARTaGENE cohort (2009–2014)	13](#_Toc229747178)

[**Supplemental Table 7** – Comparison of serum concentrations of PFOS and PFNA (µg/L) across sociodemographic, lifestyle, and reproductive history characteristics among adult females in the CARTaGENE cohort (2009–2014)	15](#_Toc229747179)

[**Supplemental Table 8** – Comparison of serum concentrations of PFOA and PFHxS (µg/L) across sociodemographic, lifestyle, and reproductive history characteristics among adult females in the CARTaGENE cohort (2009–2014)	17](#_Toc229747180)

[**Supplemental Table 9** – Comparison of serum concentrations of PFDA and PFUnA (µg/L) across sociodemographic, lifestyle, and reproductive history characteristics among adult females in the CARTaGENE cohort (2009–2014)	19](#_Toc229747181)

[**Supplemental Table** **10** – Comparison of serum concentrations of PFHpS and MeFOSAA (µg/L) across sociodemographic, lifestyle, and reproductive history characteristics among adult females in the CARTaGENE cohort (2009–2014)	21](#_Toc229747182)

[**Supplemental Table** **11** – Mean percent differences (95% CIs) in PFAS serum concentrations with each one-year increase in age stratified by number of pregnancies (parity) among adult females in the CARTaGENE cohort (2009–2014).	23](#_Toc229747183)

[*Bolded values represent coefficients and 95%CIs with p < 0.05*	23](#_Toc229747184)

[**Supplemental Figure 1** – Correlation heatmap for serum PFAS detected in > 60% of adult females in the CARTaGENE cohort (2009–2014).	24](#_Toc229747185)

[**Supplemental Figure 3** – Geometric mean (95% CI) PFOS serum concentrations by age category across parity groups among adult females in the CARTaGENE cohort (2009–2014).	26](#_Toc229747186)

[**Supplemental Figure 4** – Geometric mean (95% CI) PFNA serum concentrations by age category across parity groups among adult females in the CARTaGENE cohort (2009–2014).	27](#_Toc229747187)

[**Supplemental Figure 5** – Geometric mean (95% CI) PFOA serum concentrations by age category across parity groups among adult females in the CARTaGENE cohort (2009–2014).	28](#_Toc229747188)

[**Supplemental Figure 6** – Geometric mean (95% CI) PFHxS serum concentrations by age category across parity groups among adult females in the CARTaGENE cohort (2009–2014).	29](#_Toc229747189)

[**Supplemental Figure 7** – Geometric mean (95% CI) PFDA serum concentrations by age category across parity groups among adult females in the CARTaGENE cohort (2009–2014).	30](#_Toc229747190)

[**Supplemental Figure 8** – Geometric mean (95% CI) PFUnA serum concentrations by age category across parity groups among adult females in the CARTaGENE cohort (2009–2014).	31](#_Toc229747191)

[**Supplemental Figure 9** – Geometric mean (95% CI) PFHpS serum concentrations by age category across parity groups among adult females in the CARTaGENE cohort (2009–2014).	32](#_Toc229747192)

[**Supplemental Figure 10** – Geometric mean (95% CI) MeFOSAA serum concentrations by age category across parity groups among adult females in the CARTaGENE cohort (2009–2014).	33](#_Toc229747193)

# **Supplemental Table 1 –** Abbreviations and full names for the 40 PFAS measured in the CARTaGENE study

| **Abbreviation** | **Name - Acid Form** | **CAS RN** |  |
| --- | --- | --- | --- |
| Perfluoroalkyl carboxylates | | | |
| PFBA | Perfluorobutanoic acid | 375-22-4 |  |
| PFPeA | Perfluoropentanoic acid | 2706-90-3 |  |
| PFHxA | Perfluorohexanoic acid | 307-24-4 |  |
| PFHpA | Perfluoroheptanoic acid | 375-85-9 |  |
| PFOA | Perfluorooctanoic acid | 335-67-1 |  |
| PFNA | Perfluorononanoic acid | 375-95-1 |  |
| PFDA | Perfluorodecanoic acid | 335-76-2 |  |
| PFUnA | Perfluoroundecanoic acid | 2058-94-8 |  |
| PFDoA | Perfluorododecanoic acid | 307-55-1 |  |
| PFTrDA | Perfluorotridecanoic acid | 72629-94-8 |  |
| PFTeDA | Perfluorotetradecanoic acid | 376-06-7 |  |
| Perfluoroalkyl sulfonates | | | |
| PFBS | Perfluorobutanesulfonic acid | 375-73-5 |  |
| PFPeS | Perfluoropentanesulfonic acid | 2706-91-4 |  |
| PFHxS | Perfluorohexanesulfonic acid | 355-46-4 |  |
| PFHpS | Perfluoroheptanesulfonic acid | 375-92-8 |  |
| PFOS | Perfluorooctanesulfonic acid | 1763-23-1 |  |
| PFNS | Perfluorononanesulfonic acid | 68259-12-1 |  |
| PFDS | Perfluorodecanesulfonic acid | 335-77-3 |  |
| PFDoS | Perfluorododecanesulfonic acid | 79780-39-5 |  |
| Fluorotelomer sulfonates | | | |
| 4:2 FTS | 4:2 fluorotelomersulfonic acid | 757124-72-4 |  |
| 6:2 FTS | 6:2 fluorotelomersulfonic acid | 27619-97-2 |  |
| 8:2 FTS | 8:2 fluorotelomersulfonic acid | 39108-34-4 |  |
| Fluorotelomer carboxylates | | | |
| 3:3 FTCA | 3:3 perfluorohexanoic acid | 356-02-5 |  |
| 5:3 FTCA | 5:3 perfluorooctanoic acid | 914637-49-3 |  |
| 7:3 FTCA | 7:3 perfluorodecanoic acid | 812-70-4 |  |
| Perfluorooctane sulfonamides | | | |
| PFOSA | Perfluorooctanesulfonamide | 754-91-6 |  |
| MeFOSA | N-Methylperfluorooctanesulfonamide | 31506-32-8 |  |
| EtFOSA | N-Ethylperfluorooctanesulfonamide | 4151-50-2 |  |
| Perfluorooctane sulfonamidoacetic acids | | | |
| MeFOSAA | N-Methylperfluorooctanesulfonamidoacetic acid | 2355-31-9 |  |
| EtFOSAA | N-Ethylperfluorooctanesulfonamidoacetic acid | 2991-50-6 |  |
| Perfluorooctane sulfonamide ethanols | | | |
| MeFOSE | N-Methylperfluorooctanesulfonamidoethanol | 24448-09-7 |  |
| EtFOSE | N-Ethylperfluorooctanesulfonamidoethanol | 1691-99-2 |  |
| Per- and polyfluoroether carboxylates | | | |
| HFPO-DA | 2,3,3,3-Tetrafluoro-2-(1,1,2,2,3,3,3-heptafluoropropoxy)propanoic acid | 13252-13-6 |  |
| ADONA | Dodecafluoro-3H-4,8-dioxanonanoic acid | 919005-14-4 |  |
| NFDHA | Perfluoro-3,6-dioxaheptanoic acid | 151772-58-6 |  |
| PFMPA | Perfluoro-3-methoxypropanoic acid | 377-73-1 |  |
| PFMBA | Perfluoro-4-methoxybutanoic acid | 863090-89-5 |  |
| Ether sulfonates | | | |
| 9Cl-PF3ONS | 9-chlorohexadecafluoro-3-oxanonane-1-sulfonic acid | 756426-58-1 |  |
| 11Cl-PF3OUdS | 11-chloroeicosafluoro-3-oxaundecane-1-sulfonic acid | 763051-92-9 |  |
| PFEESA | Perfluoro(2-ethoxyethane)sulfonic acid | 113507-82-7 |  |

# **Supplemental Table 2** – Median and interquartile range of limits of detection (µg/L) for batch-specific blank samples (139 batches)

|  | **25^th^**  **percentile** | **Median** | **75^th^**  **percentile** |
| --- | --- | --- | --- |
| **Perfluoroalkyl carboxylates** | | | |
| PFBA | 0.065 | 0.106 | 0.158 |
| PFPeA | 0.048 | 0.069 | 0.085 |
| PFHxA | 0.053 | 0.065 | 0.082 |
| PFHpA | 0.046 | 0.061 | 0.076 |
| PFOA | 0.14 | 0.175 | 0.233 |
| PFNA | 0.071 | 0.095 | 0.113 |
| PFDA | 0.073 | 0.092 | 0.117 |
| PFUnA | 0.059 | 0.07 | 0.083 |
| PFDoA | 0.051 | 0.068 | 0.081 |
| PFTrDA | 0.077 | 0.102 | 0.124 |
| PFTeDA | 0.074 | 0.094 | 0.122 |
| **Perfluoroalkyl sulfonates** | | | |
| PFBS | 0.003 | 0.005 | 0.009 |
| PFPeS | 0.004 | 0.005 | 0.009 |
| PFHxS | 0.003 | 0.005 | 0.008 |
| PFHpS | 0.003 | 0.003 | 0.004 |
| PFOS | 0.003 | 0.005 | 0.01 |
| PFNS | 0.003 | 0.005 | 0.007 |
| PFDS | 0.003 | 0.005 | 0.007 |
| PFDoS | 0.002 | 0.003 | 0.004 |
| **Fluorotelomer sulfonates** | | | |
| 4:2 FTS | 0.004 | 0.007 | 0.01 |
| 6:2 FTS | 0.013 | 0.021 | 0.052 |
| 8:2 FTS | 0.007 | 0.011 | 0.019 |
| **Fluorotelomer carboxylates** | | | |
| 3:3 FTCA | 0.035 | 0.08 | 0.127 |
| 5:3 FTCA | 0.023 | 0.054 | 0.101 |
| 7:3 FTCA | 0.037 | 0.079 | 0.233 |
| **Perfluorooctane sulfonamides** | | | |
| PFOSA | 0.002 | 0.003 | 0.005 |
| MeFOSA | 0.011 | 0.014 | 0.019 |
| EtFOSA | 0.009 | 0.012 | 0.015 |
| **Perfluorooctane sulfonamidoacetic acids** | | | |
| MeFOSAA | 0.007 | 0.008 | 0.012 |
| EtFOSAA | 0.007 | 0.011 | 0.018 |
| **Perfluorooctane sulfonamide ethanols** | | | |
| MeFOSE | 0.026 | 0.031 | 0.035 |
| EtFOSE | 0.02 | 0.022 | 0.026 |
| **Per- and polyfluoroether carboxylates** | | | |
| HFPO-DA | 0.005 | 0.007 | 0.011 |
| ADONA | 0.001 | 0.001 | 0.001 |
| NFDHA | 0.026 | 0.055 | 0.117 |
| PFMPA | 0.007 | 0.011 | 0.014 |
| PFMBA | 0.002 | 0.002 | 0.003 |
| **Ether sulfonates** | | | |
| 9Cl-PF3ONS | 0.002 | 0.003 | 0.005 |
| 11Cl-PF3OUdS | 0.004 | 0.005 | 0.008 |
| PFEESA | 0.001 | 0.001 | 0.002 |

# **Supplemental Table 3** – Percent recovery of spiked surrogate standards in blank samples from 139 batches in the CARTaGENE study

| Surrogate standards | Corresponding native compounds | Surrogate standard concentration (µg/L) | Percent recovery | | |
| --- | --- | --- | --- | --- | --- |
|  |  |  | **5^th^** | **Median** | **95^th^** |
| ^13^C_2_-4:2 FTS | **4:2 FTS** | 20.2 | 79.5 | 132.0 | 179.0 |
| ^13^C_2_-6:2 FTS | **6:2 FTS** | 20 | 86.4 | 93.7 | 99.4 |
| ^13^C_2_-8:2 FTS | **8:2 FTS** | 20 | 79.4 | 95.9 | 108.1 |
| ^13^C2-PFDoA | **PFDoA** | 5 | 84.8 | 95.0 | 102.1 |
| ^13^C_2_-PFTeA and ^13^C_2_- PFDoA^1^ | **PFTrDA** | - | 82.2 | 91.4 | 99.8 |
| ^13^C_2_-PFTeDA | **PFTeDA** | 5 | 76.6 | 87.8 | 100.1 |
| ^13^C_3_-HFPO-DA | **11Cl-PF3OUdS** | 40 | 78.7 | 93.1 | 111.1 |
|  | **9Cl-PF3ONS** |  |  |  |  |
|  | **ADONA** |  |  |  |  |
|  | **HFPODA** |  |  |  |  |
| ^13^C_3_-PFBS | **PFBS** | 10 | 75.5 | 92.6 | 101.1 |
| ^13^C_3_-PFHxS | **PFHxS** | 10 | 88.4 | 96.6 | 105.0 |
|  | **PFPeS** |  |  |  |  |
| ^13^C_4_-PFBA | **PFBA** | 40 | 83.7 | 94.4 | 98.9 |
| ^13^C_4_-PFHpA | **PFHpA** | 10 | 84.5 | 93.0 | 103.1 |
| ^13^C_5_-PFHxA | **5:3 FTCA** | 10 | 76.7 | 93.8 | 102.0 |
|  | **7:3 FTCA** |  |  |  |  |
|  | **NFDHA** |  |  |  |  |
|  | **PFEESA** |  |  |  |  |
|  | **PFHxA** |  |  |  |  |
| ^13^C_5_-PFPeA | **3:3 FTCA** | 20 | 92.2 | 99.8 | 127.0 |
|  | **PFMBA** |  |  |  |  |
|  | **PFMPA** |  |  |  |  |
|  | **PFPeA** |  |  |  |  |
| ^13^C_6_-PFDA | **PFDA** | 5 | 88.0 | 96.6 | 103.0 |
| ^13^C_7_-PFUnA | **PFUnA** | 5 | 91.2 | 98.7 | 107.1 |
| ^13^C_8_-PFOA | **PFOA** | 10 | 87.6 | 95.1 | 107.1 |
| ^13^C_8_-PFOS | **PFDoS** | 10.1 | 93.3 | 101.0 | 107.1 |
|  | **PFDS** |  |  |  |  |
|  | **PFHpS** |  |  |  |  |
|  | **PFNS** |  |  |  |  |
|  | **PFOS** |  |  |  |  |
| ^13^C_8_-PFOSA | **PFOSA** | 10 | 93.4 | 108.0 | 118.0 |
| ^13^C_9_-PFNA | **PFNA** | 5 | 89.7 | 98.3 | 104.0 |
| D_3_-N-MeFOSA | **MeFOSA** | 10 | 78.7 | 93.7 | 120.3 |
| D_3_-N-MeFOSAA | **MeFOSAA** | 20 | 77.2 | 92.0 | 123.4 |
| D_5_-N-EtFOSA | **EtFOSA** | 10 | 78.6 | 92.0 | 124.0 |
| D_5_-N-EtFOSAA | **EtFOSAA** | 20 | 75.6 | 91.6 | 121.3 |
| D_7_-N-MeFOSE | **MeFOSE** | 100 | 96.0 | 112.0 | 140.1 |
| D_9_-N-EtFOSE | **EtFOSE** | 100 | 109.0 | 132.0 | 169.1 |

# **Supplemental Table 4** – Percent recovery of native per- and polyfluoroalkyl substances from two spiked reference serum samples from 139 batches in CARTaGENE study

|  | Low spike | | | | | Mid-level spike | | | | |
| --- | --- | --- | --- | --- | --- | --- | --- | --- | --- | --- |
|  | **Concentration (µg/L)** | **Percent recovery** | | | **Inter-assay CV** | **Concentration (µg/L)** | **Percent recovery** | | | **Inter-assay CV** |
|  |  | **5^th^**  **Percentile** | **Median** | **95^th^ Percentile** |  |  | **5^th^**  **Percentile** | **Median** | **95^th^ Percentile** |  |
| 3:3 FTCA | 6.4 | 10.6 | 18.3 | 30.5 | 33.4 | 20.0 | 10.6 | 18.3 | 37.3 | 38.9 |
| 5:3 FTCA | 40.0 | 70.7 | 91.5 | 117.0 | 16.2 | 125.0 | 73.7 | 89.0 | 113.0 | 14.1 |
| 7:3 FTCA | 40.0 | 80.8 | 92.6 | 119.0 | 13.0 | 125.0 | 69.4 | 84.1 | 107.0 | 14.4 |
| 4:2 FTS | 6.4 | 85.5 | 95.8 | 135.0 | 16.2 | 20.0 | 73.5 | 92.3 | 126.0 | 17.9 |
| PFDoS | 1.6 | 84.2 | 95.9 | 103.0 | 5.9 | 5.0 | 78.9 | 89.9 | 99.9 | 7.3 |
| PFMPA | 3.2 | 76.9 | 97.6 | 104.0 | 9.8 | 10.0 | 68.9 | 88.9 | 99.0 | 10.7 |
| PFMBA | 1.6 | 83.9 | 97.7 | 106.0 | 6.6 | 5.0 | 76.9 | 90.1 | 100.0 | 7.9 |
| PFPeA | 3.2 | 84.3 | 99.8 | 105.0 | 6.6 | 10.0 | 77.3 | 93.9 | 100.0 | 7.9 |
| PFNS | 1.6 | 89.7 | 99.9 | 108.0 | 5.5 | 5.0 | 83.7 | 94.1 | 103.0 | 6.6 |
| PFEESA | 1.6 | 88.3 | 101.0 | 127.0 | 12.4 | 5.0 | 80.3 | 94.1 | 120.0 | 12.9 |
| PFHxS | 1.6 | 91.8 | 101.0 | 108.0 | 5.2 | 6.4 | 83.7 | 95.3 | 103.0 | 6.3 |
| PFHxA | 1.6 | 88.8 | 101.0 | 122.0 | 11.8 | 5.0 | 80.0 | 95.4 | 111.0 | 10.7 |
| EtFOSAA | 1.6 | 91.3 | 102.0 | 111.0 | 5.9 | 5.0 | 75.5 | 90.7 | 109.0 | 10.4 |
| EtFOSE | 16.0 | 78.0 | 102.0 | 122.0 | 12.2 | 50.0 | 77.6 | 94.4 | 103.0 | 9.1 |
| PFBA | 6.4 | 95.1 | 102.0 | 107.0 | 3.8 | 20.0 | 83.7 | 95.5 | 102.0 | 5.7 |
| 9Cl-PF3ONS | 6.4 | 89.4 | 102.0 | 117.0 | 11.6 | 20.0 | 79.3 | 97.1 | 111.0 | 11.2 |
| MeFOSAA | 1.6 | 90.2 | 103.0 | 115.0 | 6.9 | 5.0 | 69.3 | 90.5 | 109.0 | 13.5 |
| 11Cl-PF3OUdS | 6.4 | 84.8 | 103.0 | 121.0 | 13.9 | 20.0 | 77.1 | 96.6 | 119.0 | 12.7 |
| EtFOSA | 4.5 | 97.3 | 104.0 | 111.0 | 3.9 | 14.0 | 81.9 | 94.1 | 107.0 | 8.9 |
| MeFOSE | 16.0 | 95.2 | 104.0 | 110.0 | 4.5 | 50.0 | 84.4 | 95.3 | 102.0 | 5.9 |
| PFOSA | 1.6 | 98.7 | 104.0 | 112.0 | 3.5 | 5.0 | 85.8 | 97.4 | 106.0 | 6.3 |
| PFOA | 1.6 | 96.4 | 104.0 | 114.0 | 4.8 | 6.4 | 87.1 | 101.5 | 116.0 | 8.7 |
| PFHpS | 1.6 | 95.3 | 105.0 | 115.0 | 5.4 | 5.0 | 83.8 | 96.9 | 109.0 | 8.1 |
| HFPO-DA | 6.4 | 90.5 | 105.0 | 115.0 | 11.0 | 20.0 | 75.6 | 97.4 | 114.0 | 12.4 |
| PFDA | 1.6 | 94.5 | 105.0 | 113.0 | 5.2 | 5.0 | 87.8 | 99.2 | 114.0 | 7.7 |
| ADONA | 6.4 | 86.4 | 105.0 | 124.0 | 13.1 | 20.0 | 75.3 | 99.6 | 117.0 | 13.5 |
| PFPeS | 1.6 | 95.6 | 106.0 | 115.0 | 5.5 | 5.0 | 85.1 | 97.6 | 108.0 | 7.5 |
| PFDS | 1.6 | 99.4 | 106.0 | 112.0 | 3.9 | 5.0 | 89.1 | 99.0 | 109.0 | 6.0 |
| NFDHA | 3.2 | 77.9 | 106.0 | 309.0 | 54.8 | 10.0 | 63.7 | 101.0 | 290.0 | 56.8 |
| PFTeDA | 1.6 | 98.9 | 106.0 | 115.0 | 4.3 | 5.0 | 90.8 | 104.0 | 114.0 | 6.6 |
| PFDoA | 1.3 | 99.3 | 107.0 | 117.0 | 5.3 | 4.1 | 85.8 | 100.0 | 116.0 | 8.8 |
| PFUnA | 1.6 | 101.0 | 107.0 | 114.0 | 4.1 | 5.0 | 88.7 | 101.0 | 113.0 | 7.4 |
| PFNA | 1.6 | 101.0 | 107.0 | 115.0 | 3.8 | 5.6 | 92.1 | 105.0 | 116.0 | 6.9 |
| PFOS | 2.3 | 101.0 | 108.0 | 121.0 | 5.5 | 8.3 | 65.4 | 79.7 | 108.0 | 16.7 |
| MeFOSA | 1.6 | 100.0 | 108.0 | 119.0 | 5.2 | 5.0 | 81.9 | 100.0 | 111.0 | 8.7 |
| PFHpA | 1.6 | 93.8 | 108.0 | 117.0 | 7.9 | 5.0 | 85.0 | 101.0 | 113.0 | 9.8 |
| PFTrDA | 1.6 | 100.0 | 108.0 | 116.0 | 4.3 | 5.0 | 92.0 | 103.0 | 113.0 | 7.0 |
| PFBS | 1.6 | 86.8 | 109.0 | 122.0 | 10.8 | 5.0 | 77.7 | 102.0 | 116.0 | 12.0 |
| 6:2 FTS | 5.8 | 105.0 | 116.0 | 144.0 | 11.7 | 18.0 | 93.7 | 107.0 | 144.0 | 13.0 |
| 8:2 FTS | 5.4 | 103.0 | 118.0 | 132.0 | 7.2 | 16.9 | 92.8 | 107.0 | 125.0 | 9.0 |

*Sorted by low-to-high for median percent recovery in the low spike*

# **Supplemental Table 5** – PFAS concentrations in clean straws following rinses using 50% formic acid and basic methanol. Results represent the average concentration of analytes in three batches of straws for each type of rinse.

|  | **Limit of detection (ng/g)** | **50% formic acid rinse of blank** | **Methanol rinse of blank** | **Spike concentration**  **(ng/g)** | **Percent recovery of spike** | **Second rinse following spike extraction** |
| --- | --- | --- | --- | --- | --- | --- |
| **PFBA** | 0.800 | Non-detect | Non-detect | 20.0 | 95.4 | Non-detect |
| **PFPeA** | 0.400 | Non-detect | Non-detect | 10.0 | 96.2 | Non-detect |
| **PFHxA** | 0.200 | Non-detect | Non-detect | 5.0 | 93.8 | Non-detect |
| **PFHPA** | 0.200 | Non-detect | Non-detect | 5.0 | 92.7 | Non-detect |
| **PFOA** | 0.200 | Non-detect | Non-detect | 5.0 | 102.5 | Non-detect |
| **PFNA** | 0.200 | Non-detect | Non-detect | 5.0 | 98.5 | Non-detect |
| **PFDA** | 0.200 | Non-detect | Non-detect | 5.0 | 99.9 | Non-detect |
| **PFUnA** | 0.200 | Non-detect | Non-detect | 5.0 | 97.0 | Non-detect |
| **PFDoA** | 0.160 | Non-detect | Non-detect | 4.1 | 89.1 | Non-detect |
| **PFTrDA** | 0.200 | Non-detect | Non-detect | 5.0 | 90.3 | Non-detect |
| **PFTeDA** | 0.200 | Non-detect | Non-detect | 5.0 | 90.8 | Non-detect |
| **PFBS** | 0.200 | Non-detect | Non-detect | 5.0 | 98.3 | Non-detect |
| **PFPeS** | 0.201 | Non-detect | Non-detect | 5.0 | 95.0 | Non-detect |
| **PFHxS** | 0.200 | Non-detect | Non-detect | 5.0 | 102.9 | Non-detect |
| **PFHpS** | 0.200 | Non-detect | Non-detect | 5.0 | 96.8 | Non-detect |
| **PFOS** | 0.200 | Non-detect | Non-detect | 5.0 | 113.0 | Non-detect |
| **PFNS** | 0.200 | Non-detect | Non-detect | 5.0 | 100.3 | Non-detect |
| **PFDS** | 0.200 | Non-detect | Non-detect | 5.0 | 93.4 | Non-detect |
| **PFDOS** | 0.200 | Non-detect | Non-detect | 5.0 | 82.1 | Non-detect |
| **4:2 FTS** | 0.800 | Non-detect | Non-detect | 20.0 | 95.9 | Non-detect |
| **6:2 FTS** | 0.721 | Non-detect | Non-detect | 18.0 | 97.2 | Non-detect |
| **8:2 FTS** | 0.680 | Non-detect | Non-detect | 16.9 | 95.6 | Non-detect |
| **PFOSA** | 0.200 | Non-detect | Non-detect | 5.0 | 92.6 | Non-detect |
| **MeFOSA** | 0.200 | Non-detect | Non-detect | 5.0 | 92.1 | Non-detect |
| **EtFOSA** | 0.560 | Non-detect | Non-detect | 14.0 | 85.6 | Non-detect |
| **MeFOSAA** | 0.200 | Non-detect | Non-detect | 5.0 | 97.0 | Non-detect |
| **EtFOSAA** | 0.200 | Non-detect | Non-detect | 5.0 | 91.3 | Non-detect |
| **MeFOSE** | 2.000 | Non-detect | Non-detect | 50.0 | 89.7 | Non-detect |
| **EtFOSE** | 2.000 | Non-detect | Non-detect | 50.0 | 91.3 | Non-detect |
| **HFPO-DA** | 0.800 | Non-detect | Non-detect | 20.0 | 92.4 | Non-detect |
| **ADONA** | 0.800 | Non-detect | Non-detect | 20.0 | 94.0 | Non-detect |
| **9CL-PF3ONS** | 0.802 | Non-detect | Non-detect | 20.0 | 93.0 | Non-detect |
| **11CL-PF3OUDS** | 0.801 | Non-detect | Non-detect | 20.0 | 91.6 | Non-detect |
| **3:3 FTCA** | 0.800 | Non-detect | Non-detect | 20.0 | 17.4 | Non-detect |
| **5:3 FTCA** | 5.000 | Non-detect | Non-detect | 125.0 | 60.3 | Non-detect |
| **7:3 FTCA** | 5.000 | Non-detect | Non-detect | 125.0 | 87.8 | Non-detect |
| **PFEESA** | 0.200 | Non-detect | Non-detect | 5.0 | 90.3 | Non-detect |
| **PFMPA** | 0.400 | Non-detect | Non-detect | 10.0 | 89.7 | Non-detect |
| **PFMBA** | 0.200 | Non-detect | Non-detect | 5.0 | 94.0 | Non-detect |
| **NFDHA** | 0.400 | Non-detect | Non-detect | 10.0 | 82.2 | Non-detect |

# **Supplemental Table** 6 – Comparison of serum concentrations of the sum of 7 PFAS (PFOS, PFNA, PFOA, PFHxS, PFDA, PFUnA, MeFOSAA) (µg/L) across sociodemographic, lifestyle, and reproductive history characteristics among adult females in the CARTaGENE cohort (2009–2014)

|  | **n** | **GM (95% CI)** | **Overall p-value** | **Pairwise p-value** |
| --- | --- | --- | --- | --- |
| **CARTaGENE phase** | | | | |
| A (2009–2010) | 1615 | 9.28 (9.06, 9.51) | **<.0001** |  |
| B (2013–2014) | 1159 | 6.50 (6.32, 6.69) |  |  |
| **Age (years)** | | | | |
| < 45 | 813 | 7.43 (7.17, 7.70) | **<.0001** | - |
| 45 – 49 | 1121 | 8.14 (7.89, 8.39) |  | **0.0005** |
| ≥ 50 | 840 | 8.40 (8.11, 8.71) |  | **<.0001** |
| **Below Canadian low income cutoff^1^** | | | | |
| No | 2396 | 8.11 (7.94, 8.28) | **<.0001** |  |
| Yes | 161 | 6.84 (6.31, 7.41) |  |  |
| **Level of education** | | | | |
| High school or less | 340 | 7.79 (7.36, 8.23) | 0.5571 |  |
| College diploma | 856 | 8.02 (7.75, 8.31) |  |  |
| University degree | 1098 | 7.96 (7.71, 8.21) |  |  |
| Graduate studies | 470 | 8.20 (7.82, 8.60) |  |  |
| **Race and ethnicity** | | | | |
| White | 2523 | 8.05 (7.88, 8.21) | 0.1038 |  |
| Other | 231 | 7.59 (7.09, 8.12) |  |  |
| **Country of birth** | | | | |
| Canada | 2475 | 7.98 (7.82, 8.15) | 0.577 |  |
| Other | 299 | 8.13 (7.66, 8.63) |  |  |
| **Time living in Canada (years)** | | | | |
| < 15 | 115 | 6.97 (6.33, 7.67) | **0.004** |  |
| ≥ 15 | 2657 | 8.05 (7.89, 8.21) |  |  |
| **Body mass index (kg/m^2^)** | | | | |
| < 25 | 1250 | 8.51 (8.27, 8.76) | **<.0001** | - |
| 25 – 29 | 765 | 7.98 (7.68, 8.28) |  | **0.021** |
| ≥ 30 | 514 | 7.57 (7.24, 7.92) |  | **<.0001** |
| **Smoking status** | | | | |
| Daily/occasional smoker | 326 | 8.17 (7.72, 8.65) | 0.4252 |  |
| Former smoker | 1002 | 8.09 (7.83, 8.36) |  |  |
| Never smoker | 1433 | 7.90 (7.69, 8.12) |  |  |
| **Physical activity (IPAQ)** | | | | |
| Low | 553 | 7.64 (7.31, 7.98) | **0.0216** | - |
| Moderate | 1113 | 8.11 (7.86, 8.36) |  | 0.0845 |
| High | 983 | 8.24 (7.97, 8.51) |  | **0.0200** |
| **Parity** | | | | |
| 0 | 628 | 9.66 (9.28, 10.1) | **<.0001** | - |
| 1 | 484 | 8.25 (7.88, 8.64) |  | **<.0001** |
| 2 | 1095 | 7.62 (7.39, 7.85) |  | **<.0001** |
| ≥ 3 | 567 | 6.95 (6.67, 7.26) |  | **<.0001** |
| **Time since last pregnancy (years)** | | | | |
| < 10 | 436 | 7.04 (6.71, 7.39) | **<.0001** | - |
| ≥ 10 – 20 | 1217 | 7.74 (7.52, 7.97) |  | **0.0064** |
| ≥ 20 | 674 | 8.20 (7.89, 8.53) |  | **<.0001** |
| Never pregnant | 441 | 9.59 (9.14, 10.1) |  | **<.0001** |
| **History of breastfeeding** | | | | |
| Yes | 1695 | 7.49 (7.31, 7.68) | **<.0001** |  |
| No | 883 | 8.76 (8.46, 9.06) |  |  |
| **Lifetime duration of breastfeeding (months)^2^** | | | | |
| 0 – 5 | 281 | 9.07 (8.58, 9.60) | 0.2263 | - |
| 6 – 11 | 272 | 8.98 (8.48, 9.50) |  | 0.99 |
| ≥ 12 | 377 | 8.55 (8.15, 8.97) |  | 0.342 |

IPAQ – International Physical Activity Questionnaire

^1^ Participants were categorized as being low income using cut-offs provided by Statistics Canada based on the size of the metropolitan area they resided in, their self-reported total annual household income, and the number of individuals in the household.

^2^ Only includes participants from Phase A who reported lifetime duration of breastfeeding

# **Supplemental Table 7** – Comparison of serum concentrations of PFOS and PFNA (µg/L) across sociodemographic, lifestyle, and reproductive history characteristics among adult females in the CARTaGENE cohort (2009–2014)

|  |  | **PFOS** |  |  | **PFNA** |  |  |
| --- | --- | --- | --- | --- | --- | --- | --- |
|  | **n** | **GM (95% CI)** | **Overall p-value** | **Pairwise p-value** | **GM (95% CI)** | **Overall p-value** | **Pairwise p-value** |
| **CARTaGENE phase** | | | | |  |  |  |
| A (2009–2010) | 1615 | 5.07 (4.92, 5.22) | **<.0001** |  | 0.77 (0.75, 0.79) | **<.0001** |  |
| B (2013–2014) | 1159 | 3.33 (3.22, 3.45) |  |  | 0.62 (0.61, 0.64) |  |  |
| **Age (years)** | | | | |  |  |  |
| < 45 | 813 | 3.94 (3.77, 4.11) | **0.0001** | - | 0.68 (0.65, 0.70) | **0.0035** | - |
| 45 – 49 | 1121 | 4.33 (4.18, 4.50) |  | **0.0031** | 0.71 (0.69, 0.73) |  | 0.128 |
| ≥ 50 | 840 | 4.47 (4.28, 4.66) |  | **0.0002** | 0.73 (0.71, 0.76) |  | **0.0024** |
| **Below Canadian low income cutoff^1^** | | | | |  |  |  |
| No | 2396 | 4.31 (4.21, 4.42) | **0.0004** |  | 0.72 (0.70, 0.73) | **<.0001** |  |
| Yes | 161 | 3.60 (3.27, 3.97) |  |  | 0.61 (0.57, 0.66) |  |  |
| **Level of education** | | | | |  |  |  |
| High school or less | 340 | 4.16 (3.89, 4.45) | 0.844 |  | 0.67 (0.63, 0.70) | **0.0043** | - |
| College diploma | 856 | 4.28 (4.10, 4.46) |  |  | 0.69 (0.67, 0.71) |  | 0.99 |
| University degree | 1098 | 4.24 (4.08, 4.40) |  |  | 0.71 (0.69, 0.74) |  | 0.1347 |
| Graduate studies | 470 | 4.32 (4.08, 4.58) |  |  | 0.75 (0.71, 0.78) |  | **0.0063** |
| **Race and ethnicity** | | | | |  |  |  |
| White | 2523 | 4.28 (4.18, 4.39) | 0.099 |  | 0.71 (0.69, 0.72) | 0.3052 |  |
| Other | 231 | 3.99 (3.67, 4.32) |  |  | 0.73 (0.69, 0.78) |  |  |
| **Country of birth** | | | | |  |  |  |
| Canada | 2475 | 4.25 (4.14, 4.35) | 0.6976 |  | 0.70 (0.69, 0.71) | **0.0034** |  |
| Other | 299 | 4.31 (4.01, 4.63) |  |  | 0.76 (0.72, 0.81) |  |  |
| **Time living in Canada (years)** | | | | |  |  |  |
| < 15 | 115 | 3.64 (3.24, 4.08) | **0.0067** |  | 0.72 (0.66, 0.79) | 0.7235 |  |
| ≥ 15 | 2657 | 4.28 (4.18, 4.39) |  |  | 0.71 (0.69, 0.72) |  |  |
| **Body mass index (kg/m^2^)** | | | | |  |  |  |
| < 25 | 1250 | 4.53 (4.38, 4.70) | **0.0017** | - | 0.74 (0.72, 0.76) | **<.0001** | - |
| 25 – 29 | 765 | 4.23 (4.04, 4.42) |  | **0.0445** | 0.71 (0.68, 0.73) |  | 0.0506 |
| ≥ 30 | 514 | 4.07 (3.85, 4.30) |  | **0.0031** | 0.67 (0.64, 0.69) |  | **<.0001** |
| **Smoking status** | | | | |  |  |  |
| Daily/occasional smoker | 326 | 4.29 (4.00, 4.59) | 0.8877 |  | 0.71 (0.67, 0.75) | 0.5797 |  |
| Former smoker | 1002 | 4.28 (4.12, 4.45) |  |  | 0.72 (0.69, 0.74) |  |  |
| Never smoker | 1433 | 4.23 (4.10, 4.37) |  |  | 0.70 (0.68, 0.72) |  |  |
| **Physical activity (IPAQ)** | | | | |  |  |  |
| Low | 553 | 4.06 (3.85, 4.28) | 0.0768 |  | 0.68 (0.66, 0.71) | 0.0962 |  |
| Moderate | 1113 | 4.33 (4.17, 4.49) |  |  | 0.71 (0.69, 0.74) |  |  |
| High | 983 | 4.36 (4.19, 4.53) |  |  | 0.72 (0.70, 0.74) |  |  |
| **Parity** | | | | |  |  |  |
| 0 | 628 | 5.26 (5.01, 5.52) | **<.0001** | - | 0.77 (0.74, 0.80) | **<.0001** | - |
| 1 | 484 | 4.45 (4.21, 4.70) |  | **<.0001** | 0.70 (0.67, 0.73) |  | **0.0174** |
| 2 | 1095 | 4.01 (3.87, 4.16) |  | **<.0001** | 0.69 (0.67, 0.71) |  | **0.0002** |
| ≥ 3 | 567 | 3.62 (3.44, 3.81) |  | **<.0001** | 0.67 (0.64, 0.70) |  | **<.0001** |
| **Time since last pregnancy (years)** | | | | |  |  |  |
| < 10 | 436 | 3.69 (3.48, 3.92) | **<.0001** | - | 0.67 (0.64, 0.70) | **0.0005** | - |
| ≥ 10 – 20 | 1217 | 4.11 (3.97, 4.26) |  | **0.0130** | 0.69 (0.68, 0.71) |  | 0.99 |
| ≥ 20 | 674 | 4.34 (4.14, 4.55) |  | **0.0002** | 0.72 (0.70, 0.75) |  | 0.0836 |
| Never pregnant | 441 | 5.23 (4.93, 5.54) |  | **<.0001** | 0.76 (0.73, 0.80) |  | **0.0009** |
| **History of breastfeeding** | | | | |  |  |  |
| Yes | 1695 | 3.92 (3.81, 4.04) | **<.0001** |  | 0.70 (0.68, 0.71) | 0.0845 |  |
| No | 883 | 4.75 (4.56, 4.95) |  |  | 0.72 (0.70, 0.74) |  |  |
| **Lifetime duration of breastfeeding (months)^2^** | | | | |  |  |  |
| 0 – 5 | 281 | 4.88 (4.56, 5.22) | 0.195 |  | 0.77 (0.73, 0.82) | 0.7653 |  |
| 6 – 11 | 272 | 4.91 (4.59, 5.26) |  |  | 0.75 (0.71, 0.79) |  |  |
| ≥ 12 | 377 | 4.57 (4.31, 4.84) |  |  | 0.760 (0.73, 0.8) |  |  |

IPAQ – International Physical Activity Questionnaire

^1^ Participants were categorized as being low income using cut-offs provided by Statistics Canada based on the size of the metropolitan area they resided in, their self-reported total annual household income, and the number of individuals in the household.

^2^ Only includes participants from Phase A who reported lifetime duration of breastfeeding

# **Supplemental Table 8** – Comparison of serum concentrations of PFOA and PFHxS (µg/L) across sociodemographic, lifestyle, and reproductive history characteristics among adult females in the CARTaGENE cohort (2009–2014)

|  |  | **PFOA** |  |  | **PFHxS** |  |  |
| --- | --- | --- | --- | --- | --- | --- | --- |
|  | **n** | **GM (95% CI)** | **Overall p-value** | **Pairwise p-value** | **GM (95% CI)** | **Overall p-value** | **Pairwise p-value** |
| **CARTaGENE phase** | | | | |  |  |  |
| A (2009–2010) | 1615 | 1.79 (1.74, 1.83) | **<.0001** |  | 0.65 (0.63, 0.68) | **<.0001** |  |
| B (2013–2014) | 1159 | 1.27 (1.24, 1.31) |  |  | 0.47 (0.45, 0.49) |  |  |
| **Age (years)** | | | | |  |  |  |
| < 45 | 813 | 1.45 (1.39, 1.50) | **<.0001** | - | 0.52 (0.49, 0.55) | **0.0002** | - |
| 45 – 49 | 1121 | 1.58 (1.53, 1.63) |  | **0.0006** | 0.58 (0.55, 0.61) |  | **0.0085** |
| ≥ 50 | 840 | 1.62 (1.57, 1.68) |  | **<.0001** | 0.61 (0.57, 0.64) |  | **0.0002** |
| **Below Canadian low income cutoff^1^** | | | | |  |  |  |
| No | 2396 | 1.57 (1.54, 1.61) | **0.0018** |  | 0.58 (0.56, 0.60) | **0.0366** |  |
| Yes | 161 | 1.38 (1.27, 1.49) |  |  | 0.50 (0.44, 0.57) |  |  |
| **Level of education** | | | | |  |  |  |
| High school or less | 340 | 1.52 (1.44, 1.61) | 0.1812 |  | 0.57 (0.52, 0.62) | 0.236 |  |
| College diploma | 856 | 1.58 (1.52, 1.63) |  |  | 0.58 (0.55, 0.61) |  |  |
| University degree | 1098 | 1.52 (1.47, 1.57) |  |  | 0.55 (0.52, 0.58) |  |  |
| Graduate studies | 470 | 1.60 (1.53, 1.68) |  |  | 0.60 (0.55, 0.64) |  |  |
| **Race and ethnicity** | | | | |  |  |  |
| White | 2523 | 1.56 (1.53, 1.59) | 0.1057 |  | 0.57 (0.55, 0.59) | 0.0882 |  |
| Other | 231 | 1.47 (1.38, 1.58) |  |  | 0.52 (0.46, 0.58) |  |  |
| **Country of birth** | | | | |  |  |  |
| Canada | 2475 | 1.55 (1.52, 1.58) | 0.9544 |  | 0.57 (0.55, 0.59) | 0.7304 |  |
| Other | 299 | 1.55 (1.46, 1.64) |  |  | 0.56 (0.51, 0.62) |  |  |
| **Time living in Canada (years)** | | | | |  |  |  |
| < 15 | 115 | 1.31 (1.19, 1.44) | **0.0003** |  | 0.46 (0.39, 0.53) | **0.0049** |  |
| ≥ 15 | 2657 | 1.56 (1.53, 1.60) |  |  | 0.57 (0.56, 0.59) |  |  |
| **Body mass index (kg/m^2^)** | | | | |  |  |  |
| < 25 | 1250 | 1.65 (1.60, 1.70) | **0.0001** | - | 0.58 (0.56, 0.61) | 0.478 |  |
| 25 – 29 | 765 | 1.56 (1.50, 1.61) |  | 0.0512 | 0.57 (0.54, 0.60) |  |  |
| ≥ 30 | 514 | 1.47 (1.41, 1.54) |  | **<.0001** | 0.55 (0.51, 0.60) |  |  |
| **Smoking status** | | | | |  |  |  |
| Daily/occasional smoker | 326 | 1.62 (1.53, 1.71) | **0.0037** | - | 0.65 (0.59, 0.71) | **0.0001** | - |
| Former smoker | 1002 | 1.60 (1.55, 1.66) |  | 0.99 | 0.59 (0.56, 0.62) |  | 0.2377 |
| Never smoker | 1433 | 1.50 (1.46, 1.55) |  | 0.0656 | 0.54 (0.51, 0.56) |  | **0.0005** |
| **Physical activity (IPAQ)** | | | | |  |  |  |
| Low | 553 | 1.48 (1.42, 1.54) | **0.0102** | - | 0.53 (0.50, 0.57) | **0.0291** | - |
| Moderate | 1113 | 1.58 (1.53, 1.63) |  | **0.0407** | 0.57 (0.54, 0.60) |  | 0.4205 |
| High | 983 | 1.60 (1.55, 1.66) |  | **0.0098** | 0.60 (0.57, 0.63) |  | **0.0249** |
| **Parity** | | | | |  |  |  |
| 0 | 628 | 1.86 (1.79, 1.94) | **<.0001** | - | 0.75 (0.70, 0.80) | **<.0001** | - |
| 1 | 484 | 1.57 (1.50, 1.65) |  | **<.0001** | 0.62 (0.58, 0.67) |  | **0.0017** |
| 2 | 1095 | 1.48 (1.44, 1.53) |  | **<.0001** | 0.52 (0.49, 0.54) |  | **<.0001** |
| ≥ 3 | 567 | 1.37 (1.31, 1.43) |  | **<.0001** | 0.47 (0.44, 0.50) |  | **<.0001** |
| **Time since last pregnancy (years)** | | | | |  |  |  |
| < 10 | 436 | 1.33 (1.27, 1.40) | **<.0001** | - | 0.49 (0.45, 0.53) | **<.0001** | - |
| ≥ 10 – 20 | 1217 | 1.51 (1.47, 1.56) |  | **<.0001** | 0.54 (0.51, 0.56) |  | 0.1600 |
| ≥ 20 | 674 | 1.59 (1.53, 1.66) |  | **<.0001** | 0.58 (0.54, 0.62) |  | **0.0033** |
| Never pregnant | 441 | 1.87 (1.78, 1.96) |  | **<.0001** | 0.74 (0.69, 0.80) |  | **<.0001** |
| **History of breastfeeding** | | | | |  |  |  |
| Yes | 1695 | 1.46 (1.42, 1.50) | **<.0001** |  | 0.52 (0.50, 0.54) | **<.0001** |  |
| No | 883 | 1.70 (1.64, 1.76) |  |  | 0.65 (0.61, 0.69) |  |  |
| **Lifetime duration of breastfeeding (months)^2^** | | | | |  |  |  |
| 0 – 5 | 281 | 1.77 (1.68, 1.87) | 0.1256 |  | 0.62 (0.56, 0.68) | 0.3706 |  |
| 6 – 11 | 272 | 1.71 (1.62, 1.81) |  |  | 0.63 (0.58, 0.69) |  |  |
| ≥ 12 | 377 | 1.64 (1.57, 1.72) |  |  | 0.58 (0.54, 0.63) |  |  |

IPAQ – International Physical Activity Questionnaire

^1^ Participants were categorized as being low income using cut-offs provided by Statistics Canada based on the size of the metropolitan area they resided in, their self-reported total annual household income, and the number of individuals in the household.

^2^ Only includes participants from Phase A who reported lifetime duration of breastfeeding

# **Supplemental Table 9** – Comparison of serum concentrations of PFDA and PFUnA (µg/L) across sociodemographic, lifestyle, and reproductive history characteristics among adult females in the CARTaGENE cohort (2009–2014)

|  |  | **PFDA** |  |  | **PFUnA** |  |  |
| --- | --- | --- | --- | --- | --- | --- | --- |
|  | **n** | **GM (95% CI)** | **Overall p-value** | **Pairwise p-value** | **GM (95% CI)** | **Overall p-value** | **Pairwise p-value** |
| **CARTaGENE phase** | | | | |  |  |  |
| A (2009–2010) | 1615 | 0.25 (0.24, 0.26) | **0.0001** |  | 0.21 (0.20, 0.22) | **<.0001** |  |
| B (2013–2014) | 1159 | 0.22 (0.21, 0.23) |  |  | 0.15 (0.14, 0.15) |  |  |
| **Age (years)** | | | | |  |  |  |
| < 45 | 813 | 0.23 (0.22, 0.24) | 0.3150 |  | 0.17 (0.16, 0.19) | 0.3874 |  |
| 45 – 49 | 1121 | 0.24 (0.23, 0.25) |  |  | 0.18 (0.17, 0.19) |  |  |
| ≥ 50 | 840 | 0.24 (0.23, 0.25) |  |  | 0.18 (0.17, 0.20) |  |  |
| **Below Canadian low income cutoff^1^** | | | | |  |  |  |
| No | 2396 | 0.24 (0.23, 0.25) | **<.0001** |  | 0.19 (0.18, 0.19) | **<.0001** |  |
| Yes | 161 | 0.19 (0.17, 0.21) |  |  | 0.13 (0.11, 0.15) |  |  |
| **Level of education** | | | | |  |  |  |
| High school or less | 340 | 0.20 (0.19, 0.22) | **<.0001** | - | 0.14 (0.13, 0.15) | **<.0001** | - |
| College diploma | 856 | 0.23 (0.22, 0.24) |  | **0.0441** | 0.17 (0.16, 0.18) |  | **0.0048** |
| University degree | 1098 | 0.24 (0.23, 0.26) |  | **0.0003** | 0.19 (0.18, 0.20) |  | **<.0001** |
| Graduate studies | 470 | 0.26 (0.24, 0.27) |  | **<.0001** | 0.22 (0.20, 0.24) |  | **<.0001** |
| **Race and ethnicity** | | | | |  |  |  |
| White | 2523 | 0.24 (0.23, 0.24) | 0.3638 |  | 0.18 (0.18, 0.19) | 0.4663 |  |
| Other | 231 | 0.25 (0.22, 0.27) |  |  | 0.17 (0.15, 0.20) |  |  |
| **Country of birth** | | | | |  |  |  |
| Canada | 2475 | 0.23 (0.23, 0.24) | **0.0001** |  | 0.18 (0.17, 0.19) | **0.013** |  |
| Other | 299 | 0.28 (0.25, 0.30) |  |  | 0.21 (0.18, 0.23) |  |  |
| **Time living in Canada (years)** | | | | |  |  |  |
| < 15 | 115 | 0.27 (0.23, 0.30) | 0.0921 |  | 0.18 (0.15, 0.21) | 0.7121 |  |
| ≥ 15 | 2657 | 0.23 (0.23, 0.24) |  |  | 0.18 (0.18, 0.19) |  |  |
| **Body mass index (kg/m^2^)** | | | | |  |  |  |
| < 25 | 1250 | 0.26 (0.25, 0.27) | **<.0001** | - | 0.21 (0.20, 0.22) | **<.0001** |  |
| 25 – 29 | 765 | 0.23 (0.22, 0.25) |  | **0.0213** | 0.18 (0.17, 0.19) |  | **<0.0001** |
| ≥ 30 | 514 | 0.20 (0.19, 0.21) |  | **<.0001** | 0.13 (0.12, 0.15) |  | **<0.0001** |
| **Smoking status** | | | | |  |  |  |
| Daily/occasional smoker | 326 | 0.22 (0.20, 0.24) | 0.2360 |  | 0.17 (0.16, 0.19) | 0.2081 |  |
| Former smoker | 1002 | 0.24 (0.23, 0.25) |  |  | 0.19 (0.18, 0.20) |  |  |
| Never smoker | 1433 | 0.24 (0.23, 0.25) |  |  | 0.18 (0.17, 0.19) |  |  |
| **Physical activity (IPAQ)** | | | | |  |  |  |
| Low | 553 | 0.23 (0.22, 0.24) | 0.1016 |  | 0.17 (0.15, 0.18) | **0.0034** | - |
| Moderate | 1113 | 0.23 (0.22, 0.24) |  |  | 0.18 (0.17, 0.19) |  | 0.1217 |
| High | 983 | 0.25 (0.24, 0.26) |  |  | 0.19 (0.18, 0.21) |  | **0.0023** |
| **Parity** | | | | |  |  |  |
| 0 | 628 | 0.25 (0.24, 0.27) | 0.0536 |  | 0.19 (0.18, 0.21) | 0.1214 |  |
| 1 | 484 | 0.23 (0.21, 0.24) |  |  | 0.17 (0.16, 0.19) |  |  |
| 2 | 1095 | 0.24 (0.23, 0.25) |  |  | 0.18 (0.17, 0.19) |  |  |
| ≥ 3 | 567 | 0.23 (0.21, 0.24) |  |  | 0.18 (0.16, 0.19) |  |  |
| **Time since last pregnancy (years)** | | | | |  |  |  |
| < 10 | 436 | 0.24 (0.22, 0.25) | 0.3436 |  | 0.18 (0.16, 0.20) | 0.9223 |  |
| ≥ 10 – 20 | 1217 | 0.23 (0.22, 0.24) |  |  | 0.18 (0.17, 0.19) |  |  |
| ≥ 20 | 674 | 0.24 (0.23, 0.26) |  |  | 0.18 (0.17, 0.20) |  |  |
| Never pregnant | 441 | 0.24 (0.23, 0.26) |  |  | 0.18 (0.17, 0.20) |  |  |
| **History of breastfeeding** | | | | |  |  |  |
| Yes | 1695 | 0.24 (0.23, 0.25) | 0.0853 |  | 0.18 (0.18, 0.19) | 0.0912 |  |
| No | 883 | 0.23 (0.22, 0.24) |  |  | 0.17 (0.16, 0.18) |  |  |
| **Lifetime duration of breastfeeding (months)^2^** | | | | |  |  |  |
| 0 – 5 | 281 | 0.24 (0.22, 0.26) | 0.4165 |  | 0.19 (0.17, 0.21) | **0.0114** | - |
| 6 – 11 | 272 | 0.25 (0.22, 0.27) |  |  | 0.24 (0.21, 0.26) |  | **0.0123** |
| ≥ 12 | 377 | 0.26 (0.24, 0.28) |  |  | 0.22 (0.20, 0.24) |  | 0.0762 |

IPAQ – International Physical Activity Questionnaire

^1^ Participants were categorized as being low income using cut-offs provided by Statistics Canada based on the size of the metropolitan area they resided in, their self-reported total annual household income, and the number of individuals in the household.

^2^ Only includes participants from Phase A who reported lifetime duration of breastfeeding

# **Supplemental Table** **10** – Comparison of serum concentrations of PFHpS and MeFOSAA (µg/L) across sociodemographic, lifestyle, and reproductive history characteristics among adult females in the CARTaGENE cohort (2009–2014)

|  |  | **PFHpS** |  |  | **MeFOSAA** |  |  |
| --- | --- | --- | --- | --- | --- | --- | --- |
|  | **n** | **GM (95% CI)** | **Overall p-value** | **Pairwise p-value** | **GM (95% CI)** | **Overall p-value** | **Pairwise p-value** |
| **CARTaGENE phase** | | | | |  |  |  |
| A (2009–2010) | 1615 | 0.04 (0.04, 0.04) | **<.0001** |  | 0.04 (0.03, 0.04) | **<.0001** |  |
| B (2013–2014) | 1159 | 0.03 (0.02, 0.03) |  |  | 0.02 (0.02, 0.02) |  |  |
| **Age (years)** | | | | |  |  |  |
| < 45 | 813 | 0.03 (0.03, 0.03) | **0.0004** | - | 0.02 (0.02, 0.03) | **0.0099** | - |
| 45 – 49 | 1121 | 0.03 (0.03, 0.04) |  | 0.1472 | 0.03 (0.02, 0.03) |  | 0.1781 |
| ≥ 50 | 840 | 0.04 (0.04, 0.04) |  | **0.0002** | 0.03 (0.03, 0.03) |  | **0.0075** |
| **Below Canadian low income cutoff^1^** | | | | |  |  |  |
| No | 2396 | 0.03 (0.03, 0.04) | 0.3474 |  | 0.03 (0.02, 0.03) | 0.3652 |  |
| Yes | 161 | 0.03 (0.02, 0.04) |  |  | 0.02 (0.02, 0.03) |  |  |
| **Level of education** | | | | |  |  |  |
| High school or less | 340 | 0.03 (0.03, 0.04) | 0.3695 |  | 0.02 (0.02, 0.03) | 0.2965 |  |
| College diploma | 856 | 0.04 (0.03, 0.04) |  |  | 0.03 (0.02, 0.03) |  |  |
| University degree | 1098 | 0.03 (0.03, 0.04) |  |  | 0.03 (0.02, 0.03) |  |  |
| Graduate studies | 470 | 0.03 (0.03, 0.04) |  |  | 0.03 (0.02, 0.03) |  |  |
| **Race and ethnicity** | | | | |  |  |  |
| White | 2523 | 0.03 (0.03, 0.04) | 0.1405 |  | 0.03 (0.02, 0.03) | 0.0522 |  |
| Other | 231 | 0.03 (0.02, 0.04) |  |  | 0.02 (0.02, 0.03) |  |  |
| **Country of birth** | | | | |  |  |  |
| Canada | 2475 | 0.03 (0.03, 0.04) | 0.9573 |  | 0.03 (0.02, 0.03) | 0.0535 |  |
| Other | 299 | 0.03 (0.03, 0.04) |  |  | 0.02 (0.02, 0.03) |  |  |
| **Time living in Canada (years)** | | | | |  |  |  |
| < 15 | 115 | 0.03 (0.02, 0.03) | 0.0689 |  | 0.02 (0.01, 0.02) | **0.0002** |  |
| ≥ 15 | 2657 | 0.03 (0.03, 0.04) |  |  | 0.03 (0.02, 0.03) |  |  |
| **Body mass index (kg/m^2^)** | | | | |  |  |  |
| < 25 | 1250 | 0.03 (0.03, 0.04) | 0.2642 |  | 0.03 (0.03, 0.03) | **0.0462** |  |
| 25 – 29 | 765 | 0.03 (0.03, 0.04) |  |  | 0.03 (0.02, 0.03) |  |  |
| ≥ 30 | 514 | 0.04 (0.03, 0.04) |  |  | 0.02 (0.02, 0.03) |  |  |
| **Smoking status** | | | | |  |  |  |
| Daily/occasional smoker | 326 | 0.04 (0.03, 0.05) | **0.0337** | - | 0.03 (0.02, 0.03) | 0.4238 |  |
| Former smoker | 1002 | 0.04 (0.03, 0.04) |  | 0.6859 | 0.02 (0.02, 0.03) |  |  |
| Never smoker | 1433 | 0.03 (0.03, 0.03) |  | 0.0524 | 0.03 (0.02, 0.03) |  |  |
| **Physical activity (IPAQ)** | | | | |  |  |  |
| Low | 553 | 0.03 (0.03, 0.04) | 0.4462 |  | 0.02 (0.02, 0.03) | **0.0332** | - |
| Moderate | 1113 | 0.03 (0.03, 0.04) |  |  | 0.03 (0.03, 0.03) |  | **0.0317** |
| High | 983 | 0.04 (0.03, 0.04) |  |  | 0.03 (0.02, 0.03) |  | 0.6046 |
| **Parity** | | | | |  |  |  |
| 0 | 628 | 0.05 (0.05, 0.06) | **<.0001** | - | 0.03 (0.03, 0.03) | **0.0061** | - |
| 1 | 484 | 0.04 (0.03, 0.05) |  | **0.0343** | 0.03 (0.02, 0.03) |  | 0.437 |
| 2 | 1095 | 0.03 (0.03, 0.03) |  | **<.0001** | 0.02 (0.02, 0.03) |  | **0.0076** |
| ≥ 3 | 567 | 0.03 (0.02, 0.03) |  | **<.0001** | 0.02 (0.02, 0.03) |  | **0.019** |
| **Time since last pregnancy (years)** | | | | |  |  |  |
| < 10 | 436 | 0.03 (0.02, 0.03) | **<.0001** | - | 0.02 (0.02, 0.03) | **0.0196** | - |
| ≥ 10 – 20 | 1217 | 0.03 (0.03, 0.03) |  | 0.99 | 0.03 (0.02, 0.03) |  | 0.6462 |
| ≥ 20 | 674 | 0.04 (0.03, 0.04) |  | **0.004** | 0.03 (0.02, 0.03) |  | 0.99 |
| Never pregnant | 441 | 0.05 (0.04, 0.06) |  | **<.0001** | 0.03 (0.03, 0.04) |  | **0.0123** |
| **History of breastfeeding** | | | | |  |  |  |
| Yes | 1695 | 0.03 (0.03, 0.03) | **<.0001** |  | 0.03 (0.02, 0.03) | 0.2156 |  |
| No | 883 | 0.04 (0.04, 0.05) |  |  | 0.03 (0.02, 0.03) |  |  |
| **Lifetime duration of breastfeeding (months)^2^** | | | | |  |  |  |
| 0 – 5 | 281 | 0.04 (0.04, 0.05) | 0.0521 |  | 0.03 (0.03, 0.04) | 0.3411 |  |
| 6 – 11 | 272 | 0.03 (0.03, 0.04) |  |  | 0.03 (0.03, 0.04) |  |  |
| ≥ 12 | 377 | 0.03 (0.03, 0.04) |  |  | 0.04 (0.03, 0.04) |  |  |

IPAQ – International Physical Activity Questionnaire

^1^ Participants were categorized as being low income using cut-offs provided by Statistics Canada based on the size of the metropolitan area they resided in, their self-reported total annual household income, and the number of individuals in the household.

^2^ Only includes participants from Phase A who reported lifetime duration of breastfeeding

# **Supplemental Table** **11** – Mean percent differences (95% CIs) in PFAS serum concentrations with each one-year increase in age stratified by number of pregnancies (parity) among adult females in the CARTaGENE cohort (2009–2014).

|  | Nulliparous  (n = 628) | 1 prior pregnancy  (n = 484) | 2 prior pregnancies  (n = 1096) | ≥ 3 prior pregnancies  (n = 567) |
| --- | --- | --- | --- | --- |
| Σ7PFAS | 0.1 (-0.9, 1.0) | **1.7 (0.6, 2.7)** | **1.4 (0.7, 2.1)** | **2.4 (1.5, 3.4)** |
| PFOS | -0.3 (-1.4, 0.9) | **1.8 (0.6, 3.1)** | **1.6 (0.8, 2.5)** | **2.5 (1.4, 3.6)** |
| PFNA | 0.8 (-0.1, 1.7) | 0.9 (-0.1, 1.9) | **0.8 (0.1, 1.4)** | **1.2 (0.4, 2.1)** |
| PFOA | 0.5 (-0.9, 1.0) | **1.5 (0.5, 2.5)** | **1.2 (0.5, 1.9)** | **2.5 (1.6, 3.5)** |
| PFHxS | 1.0 (-0.5, 2.6) | 1.3 (-0.3, 3.1) | **2.1 (1.0, 3.2)** | **3.4 (1.9, 4.9)** |
| PFDA | -0.3 (-1.8, 1.1) | 0.6 (-1.0, 2.1) | 0.4 (-0.6, 1.4) | 1.3 (-0.0, 2.6) |
| PFUnA | 0.0 (-1.7, 1.8) | 1.8 (-0.0, 3.8) | -0.2 (-1.4, 1.0) | 0.8 (-0.8, 2.5) |
| PFHpS | 2.0 (-1.3, 5.3) | **3.7 (0.2, 7.3)** | **3.7 (1.4, 6.1)** | **5.9 (2.8, 9.1)** |
| MeFOSAA | 2.6 (-0.4, 5.6) | **5.0 (1.8, 8.3)** | **2.3 (0.3, 4.4)** | **2.3 (-0.3, 5.1)** |

# *Bolded values represent coefficients and 95%CIs with p < 0.05*

# **Supplemental Figure 1** – Correlation heatmap for serum PFAS detected in > 60% of adult females in the CARTaGENE cohort (2009–2014).

**Supplemental Figure 2** – Detection frequencies, geometric means (GM) and 95^th^ percentile concentrations of PFAS measured in the CARTaGENE cohort (Phase A: 2009–2010 and Phase B: 2013–2014) and females aged 40–59 from the 2010–2011 Canadian Health Measures Survey (CHMS). 95^th^ percentile for PFHxS from the CHMS is not presented as per Statistics Canada’s suppression requirements for confidentiality.

Detection frequency (%)

Concentration (µg/L)

|  | CHMS cycle 2 detection | CARTaGENE full sample detection | CARTaGENE Phase A detection | CARTaGENE Phase B detection |
| --- | --- | --- | --- | --- |
| PFOA | 100 | 99.6 | 99.9 | 99.1 |
| PFOS | 99.4 | 100 | 100 | 100 |
| PFHxS | 97.5 | 99.6 | 99.9 | 99.1 |
| PFNA | 99.4 | 99.8 | 99.8 | 99.8 |
| PFDA | 76.6 | 90 | 90 | 90 |
| PFUnA | 76.6 | 85 | 89.6 | 78.6 |
| PFBA | 0 | 67 | 75 | 54 |
| PFHxA | 0.63 | 31 | 37 | 23 |
| PFBS | 0 | 29 | 35 | 20 |

|  | CHMS cycle 2 GM | CHMS cycle 2 95th percentile |
| --- | --- | --- |
| 0.73 | 2.1 | 4.1 |
| 1.73 | 5.6 | 16 |
| 2.73 | 1.5 |  |
| 3.73 | 0.81 | 1.6 |
| 4.73 | 0.16 | 0.49 |
| 5.73 | 0.11 | 0.39 |

|  | CARTaGENE full sample GM | CARTaGENE full sample 95th percentile |
| --- | --- | --- |
| 0.9 | 1.552 | 3.512 |
| 1.9 | 4.253 | 11.33 |
| 2.9 | 0.568 | 2.173 |
| 3.9 | 0.707 | 1.456 |
| 4.9 | 0.236 | 0.616 |
| 5.9 | 0.18 | 0.607 |
| 6.9 | 0.033 | 0.202 |
| 7.9 |  | 0.12 |
| 8.9 |  | 0.062 |

|  | Phase A GM | Phase A 95th percentile |
| --- | --- | --- |
| 1.09 | 1.788 | 3.922 |
| 2.09 | 5.069 | 12.25 |
| 3.09 | 0.653 | 2.264 |
| 4.09 | 0.773 | 1.538 |
| 5.09 | 0.246 | 0.674 |
| 6.09 | 0.209 | 0.655 |
| 7.09 | 0.024 | 0.257 |
| 8.09 |  | 0.123 |
| 9.09 |  | 0.108 |

|  | Phase B GM | Phase B 95th percentile |
| --- | --- | --- |
| 1.27 | 1.275 | 2.744 |
| 2.27 | 3.331 | 8.98 |
| 3.27 | 0.469 | 1.859 |
| 4.27 | 0.624 | 1.3 |
| 5.27 | 0.221 | 0.563 |
| 6.27 | 0.145 | 0.524 |
| 7.27 | 0.053 | 0.251 |
| 8.27 |  | 0.117 |
| 9.27 |  | 0.026 |

# **Supplemental Figure 3** – Geometric mean (95% CI) PFOS serum concentrations by age category across parity groups among adult females in the CARTaGENE cohort (2009–2014).

| Age categories by parity | PFOS (µg/L) |
| --- | --- |
| 0 < 45 | 5.3503 |
| 0 45 – 49 | 5.3238 |
| 0 ≥ 50 | 5.07465 |
| 1 < 45 | 4.03604 |
| 1 45 – 49 | 4.47857 |
| 1 ≥ 50 | 4.96628 |
| 2 < 45 | 3.68091 |
| 2 45 – 49 | 4.04862 |
| 2 ≥ 50 | 4.30555 |
| 3 < 45 | 3.07729 |
| 3 45 – 49 | 3.71961 |
| 3 ≥ 50 | 3.96397 |

# **Supplemental Figure 4** – Geometric mean (95% CI) PFNA serum concentrations by age category across parity groups among adult females in the CARTaGENE cohort (2009–2014).

| Age categories by parity | PFNA (µg/L) |
| --- | --- |
| 0 < 45 | 0.745573 |
| 0 45 – 49 | 0.778481 |
| 0 ≥ 50 | 0.774093 |
| 1 < 45 | 0.687648 |
| 1 45 – 49 | 0.67928 |
| 1 ≥ 50 | 0.758121 |
| 2 < 45 | 0.66553 |
| 2 45 – 49 | 0.69853 |
| 2 ≥ 50 | 0.716883 |
| 3 < 45 | 0.615501 |
| 3 45 – 49 | 0.671092 |
| 3 ≥ 50 | 0.713765 |

# **Supplemental Figure 5** – Geometric mean (95% CI) PFOA serum concentrations by age category across parity groups among adult females in the CARTaGENE cohort (2009–2014).

| Age categories by parity | PFOA µ(g/L) |
| --- | --- |
| 0 < 45 | 1.8266 |
| 0 45 – 49 | 1.89691 |
| 0 ≥ 50 | 1.83481 |
| 1 < 45 | 1.46546 |
| 1 45 – 49 | 1.56358 |
| 1 ≥ 50 | 1.73087 |
| 2 < 45 | 1.39766 |
| 2 45 – 49 | 1.49089 |
| 2 ≥ 50 | 1.56096 |
| 3 < 45 | 1.15266 |
| 3 45 – 49 | 1.42316 |
| 3 ≥ 50 | 1.49365 |

# **Supplemental Figure 6** – Geometric mean (95% CI) PFHxS serum concentrations by age category across parity groups among adult females in the CARTaGENE cohort (2009–2014).

| Age categories by parity | PFHxS (µg/L) |
| --- | --- |
| 0 < 45 | 0.685173 |
| 0 45 – 49 | 0.758479 |
| 0 ≥ 50 | 0.790251 |
| 1 < 45 | 0.580191 |
| 1 45 – 49 | 0.608945 |
| 1 ≥ 50 | 0.691339 |
| 2 < 45 | 0.475373 |
| 2 45 – 49 | 0.521235 |
| 2 ≥ 50 | 0.556273 |
| 3 < 45 | 0.381191 |
| 3 45 – 49 | 0.485723 |
| 3 ≥ 50 | 0.516447 |

# **Supplemental Figure 7** – Geometric mean (95% CI) PFDA serum concentrations by age category across parity groups among adult females in the CARTaGENE cohort (2009–2014).

| Age categories by parity | PFDA (µg/L) |
| --- | --- |
| 0 < 45 | 0.262404 |
| 0 45 – 49 | 0.250523 |
| 0 ≥ 50 | 0.246504 |
| 1 < 45 | 0.214813 |
| 1 45 – 49 | 0.238284 |
| 1 ≥ 50 | 0.230515 |
| 2 < 45 | 0.22524 |
| 2 45 – 49 | 0.240887 |
| 2 ≥ 50 | 0.238128 |
| 3 < 45 | 0.213221 |
| 3 45 – 49 | 0.222292 |
| 3 ≥ 50 | 0.244537 |

# **Supplemental Figure 8** – Geometric mean (95% CI) PFUnA serum concentrations by age category across parity groups among adult females in the CARTaGENE cohort (2009–2014).

| Age categories by parity | PFUnA (µg/L) |
| --- | --- |
| 0 < 45 | 0.192337 |
| 0 45 – 49 | 0.201408 |
| 0 ≥ 50 | 0.186111 |
| 1 < 45 | 0.158344 |
| 1 45 – 49 | 0.167146 |
| 1 ≥ 50 | 0.194928 |
| 2 < 45 | 0.175221 |
| 2 45 – 49 | 0.189 |
| 2 ≥ 50 | 0.173458 |
| 3 < 45 | 0.171223 |
| 3 45 – 49 | 0.169289 |
| 3 ≥ 50 | 0.192239 |

# **Supplemental Figure 9** – Geometric mean (95% CI) PFHpS serum concentrations by age category across parity groups among adult females in the CARTaGENE cohort (2009–2014).

| Age categories by parity | PFHpS (µg/L) |
| --- | --- |
| 0 < 45 | 0.0467741 |
| 0 45 – 49 | 0.0499965 |
| 0 ≥ 50 | 0.059763 |
| 1 < 45 | 0.0340238 |
| 1 45 – 49 | 0.0379209 |
| 1 ≥ 50 | 0.0465346 |
| 2 < 45 | 0.0244457 |
| 2 45 – 49 | 0.0278151 |
| 2 ≥ 50 | 0.0341085 |
| 3 < 45 | 0.0181967 |
| 3 45 – 49 | 0.0259271 |
| 3 ≥ 50 | 0.0334161 |

# **Supplemental Figure 10** – Geometric mean (95% CI) MeFOSAA serum concentrations by age category across parity groups among adult females in the CARTaGENE cohort (2009–2014).

| Age categories by parity | MeFOSAA (µg/L) |
| --- | --- |
| 0 < 45 | 0.0266238 |
| 0 45 – 49 | 0.0318325 |
| 0 ≥ 50 | 0.0348321 |
| 1 < 45 | 0.0245591 |
| 1 45 – 49 | 0.0213385 |
| 1 ≥ 50 | 0.0377067 |
| 2 < 45 | 0.0216739 |
| 2 45 – 49 | 0.0251579 |
| 2 ≥ 50 | 0.0255406 |
| 3 < 45 | 0.0197491 |
| 3 45 – 49 | 0.0260517 |
| 3 ≥ 50 | 0.0248096 |
